# Proteomic and phosphorylated proteomic landscape of injured lung in juvenile septic rats with therapeutic application of umbilical cord mesenchymal stem cells

**DOI:** 10.3389/fimmu.2022.1034821

**Published:** 2022-10-21

**Authors:** Hongwu Wang, Junlin Luo, Aijia Li, Xing Su, Chuiqin Fang, Lichun Xie, Yi Wu, Feiqiu Wen, Yufeng Liu, Tianyou Wang, Yong Zhong, Lian Ma

**Affiliations:** ^1^ Department of Pediatrics, The Second Affiliated Hospital of Shantou University Medical College, Shantou, China; ^2^ Department of Hematology and Oncology, Shenzhen Children's Hospital of China Medical University, Shenzhen, China; ^3^ Department of Hematology and Oncology, Shenzhen Children's Hospital, Shenzhen, China; ^4^ Department of Pediatrics, The Third Affiliated Hospital of Guangzhou Medical University (The Women and Children’s Medical Hospital of Guangzhou Medical University), Guangzhou, China; ^5^ Department of Hematology and Oncology, Shenzhen Public Service Platform of Molecular Medicine in Pediatric Hematology and Oncology, Shenzhen, China; ^6^ Department of Pediatrics, The First Affiliated Hospital of Zhengzhou University, Zhengzhou, China; ^7^ Department of Hematology and Oncology, Beijing Children’s Hospital, Capital Medical University, Beijing, China; ^8^ Department of Pediatrics, The Southeast General Hospital of Dongguan, Dongguan, China

**Keywords:** sepsis, pediatric, rat, cecal contents, umbilical cord mesenchymal stem cells, lung injury, proteomics, protein phosphorylation

## Abstract

Acute lung injury (ALI) is the most common complication of sepsis. Intravenous injection of HUMSCs can regulate the level of circulating endothelial cytokines and alleviate lung injury in juvenile septic rats. In this study, we performed proteomic and phosphorylated proteomic analysis of lung tissue of juvenile septic rats after Human Umbilical Cord Mesenchymal Stem Cells (HUMSCs) intervention for the first time, and screened the potential proteins and pathways of HUMSCs for therapeutic effect. The 4D proteome quantitative technique was used to quantitatively analyze the lung tissues of septic rats 24 hours (3 biological samples) and 24 hours after HUMSCs intervention (3 biological samples). A total of 213 proteins were identified as differentially expressed proteins, and 971 phosphorylation sites changed significantly. Based on the public database, we analyzed the functional enrichment of these proteins and phosphorylated proteins. In addition, Tenascin-C may be the key differential protein and ECM receptor interaction pathway may be the main signal pathway by using various algorithms to analyze the protein-protein interaction network. Phosphorylation analysis showed that tight junction pathway was closely related to immune inflammatory reaction, and EGFR interacted most, which may be the key differential phosphorylated protein. Finally, 123 conserved motifs of serine phosphorylation site (pS) and 17 conserved motifs of threonine (pT) phosphorylation sites were identified by motif analysis of phosphorylation sites. Results from proteomics and phosphorylated proteomics, the potential new therapeutic targets of HUMSCs in alleviating lung injury in juvenile septic rats were revealed.

## Introduction

Lung is the main target organ of multiple organ dysfunction in sepsis (1). It has been reported in the literature that more than 50% of septic patients will have septic-induced acute lung injury (ALI) (2). At present, the main treatment for sepsis is still glucocorticoid, gamma globulin and symptomatic support treatment. There is no effective drug for septic lung injury except lung protective ventilation and fluid management strategy (3).

MSCs (Mesenchymal Stem Cells, MSCs) have immunomodulatory and paracrine functions (4, 5). In recent years, it has been reported that MSCs intervention can promote the repair of septic ALI and play a positive role (6–8). Studies have preliminarily confirmed that MSCs can effectively inhibit the secretion of cellular inflammatory factors and improve the prognosis of COVID-19 (9). The in-depth mechanism of MSCs-based therapy for septic lung injury is still not well defined. The therapeutic effect is affected by many factors, among which the interaction mechanism between MSCs and lung injury is an important factor (10).

Protein phosphorylation is the most common type of post-translational modification, and more than 30% of proteins in cells are phosphorylated, which is the most common and important mechanism to regulate protein function (11). Studies have found that MSCs can improve chronic inflammatory diseases such as chronic complications of diabetes, nerve cells and spinal cord injury by regulating key signaling pathways or protein phosphorylation (12–15).

It has been reported that the phosphorylation level of nuclear factor (NF) -κB p65 in the lung tissue of septic mice decreased after the intervention of bone marrow stromal cell conditioned medium (MSC-CM), and the mechanism may be related to the inhibition of phosphorylation of NF-κB pathway by MSC-CM (16). The results showed that the intervention of HUMSCs reduced the expression of MyD88 mRNA and protein in the liver tissue of septic mice induced by cecal ligation and puncture (CLP), as well as the proportion of NFκB phosphorylation, which was closely related to the phosphorylation of MyD88-NFκB pathway (17). Pedrazza et al. found that MSCs can inhibit the activation of MAPK (mitogen-activated protein kinase, MAPK) pathway and regulate the inflammatory response in sepsis, and its mechanism may be directly related to the ability of BMMSCs to inhibit the phosphorylation of ERK, RSK and p38 in the MAPK family (18). Chen et al. showed that MSC SEV (Small Extracellular Vesicles, SEV) could improve the pulmonary microvascular permeability of ALI induced by sepsis, inhibit the pathological changes of lung tissue and the infiltration of neutrophils into lung tissue, and found that the protective effect of MSC SEV on lung may be related to its inhibition of phosphorylation of MAPK/NF-κB pathway and degradation of IκB (19).

Our study initially confirmed that HUMSCs can regulate the expression of endothelial cytokines through its immunomodulatory function, reduce inflammatory injury, and thus improve the symptoms and survival rate of juvenile septic rats. However, whether HUMSCs treatment can improve lung injury in juvenile septic rats, and which key proteins or protein phosphorylation modifications play a role still need further study.

Therefore, this study will use 4D proteome quantitative technology to quantitatively analyze the lung tissue proteome and protein phosphorylation modification group of 24 hours after sepsis (SIRs24) and 24 hours after HUMSCs intervention (MS24) in juvenile septic rats. Constructing a lung tissue-specific protein database, annotating common functions of the identified proteins, performing quantitative analysis of phosphorylation modification sites, performing difference screening according to the quantitative analysis result, performing motif analysis on the modification sites based on the difference analysis result, and performing functional classification statistical analysis on the differentially modified proteins; Based on the statistical results of different classification methods, Fisher’s exact test was used for enrichment analysis, and enrichment cluster analysis was used to compare the functional links of differentially modified proteins after HUMSCs intervention; Finally, protein interaction network (PPI) analysis was used to screen out the potential key regulatory modification proteins and reveal the potential molecular mechanism of HUMSCs to alleviate septic lung injury.

## Materials and methods

### Preparation of juvenile septic rats

Forty-one SPF grade 3-week-old SD rats were fed adaptively for one week, including 2 cecal contents donor rats, 6 normal rats (including 3 for sampling and 3 for mortality study), and 36 rats in the sepsis group of intraperitoneal injection of cecal contents solution (including 18 for sampling at different time points and 15 for mortality study). The pediatric sepsis rat model was induced by intraperitoneal injection of cecal content. When sepsis symptoms appeared after intraperitoneal injection of cecal contents (0 hour), the general condition of rats were evaluated at 3 hours, 9 hours, 24 hours, 48 hours and 72 hours after symptoms appeared. 3 rats were sacrificed at each observation time point to evaluate the sepsis model from the aspects of blood routine, biochemical indexes, immune cells, endothelial cytokines, nonspecific inflammatory factors, mortality, and pathological morphology of important organs such as lung and liver.The animal experiment was approved by the Ethics Committee of Shenzhen Topu Biological Laboratory Animal Center (Ethics Approval No.: TOP-IACUC-2021-0003).

### Preparation of HUMSCs

Primary culture of HUMSCs: The acquisition and culture of fetal umbilical cord has been approved by the Ethics Committee of the Second Affiliated Hospital of Shantou University School of Medicine (Ethical Approval No.: 2021-89). After the birth of a healthy full-term newborn by cesarean section, the fetal umbilical cord was obtained under aseptic operation, placed in a 50ml centrifuge tube containing sterile PBS, and quickly transported to the laboratory in ice cubes. Clean and disinfect the ultra-clean table operated by a single person for at least 30 min, move the umbilical cord to another 50ml centrifuge tube filled with iodophor, and soak completely for 2-3min. Rinse the umbilical cord twice with 0.9% sterile saline, peel off the two umbilical arteries, umbilical vein and amniotic membrane, tear off Walton gum (umbilical cord matrix) with tweezers, cut it into 1-3mm3 tissue pieces, then lay the plates evenly, add DMEM/F12 medium containing 10% FBS, and culture it in a 5% CO_2_ at 37 °C. After 7-14 days, cells can be observed under inverted phase contrast microscope, and cells can be changed or passaged according to the density of cell growth adherence.

Subculture of HUMSCs: When the primary cells of HUMSCs grow to 80-90% density in Petri dish under inverted phase contrast microscope, cell subculture can be carried out (taking 100 mm Petri dish as an example). Absorb and discard the old culture medium in a single-person ultra-clean table, gently wash it with sterile PBS preheated at 37 °C for 2 times, wash off dead cells, add 1 mL of EDTA trypsin containing 0.25%, shake well, put it at 37 °C, and incubate it in a 5% CO_2_ for 1-2 min. Blow with a pipette gun until the adherent cells separate and fall off to form cell suspension. The 4 mL cell suspension was transferred into a 15 mL sterile centrifuge tube and whipped evenly. The 2 mL cell suspension was sucked out and placed in a new 100 mm cell culture dish at a ratio of 1: 2 or 1: 3. Each dish was supplemented to 8-10 mL, and then placed in a CO_2_ culture dish to continue culture. When the cell density reached 90% again, cell passage could be carried out again. The third generation of HUMSCs (3×10^6^/kg) was injected into juvenile septic rats *via* tail vein.

### Experimental grouping

HUMSCs treatment group: The third generation HUMSCs (3×10^6^/kg) were injected into juvenile septic rats through tail vein; Sepsis model group: The same amount of normal saline was injected into juvenile septic rats through tail vein. In this study, the lung tissue of septic rats 24 hours after sepsis (SIRs24) and 24 hours after HUMSCs intervention (MS24) were analyzed quantitatively in proteomic and phosphorylated groups.

### Experimental materials and reagents

The materials and reagents required for sample preparation are shown in **Supplementary Table 1**.

### Protein extraction

Take out the lung tissue sample to be tested from -80 °C, weigh a proper amount of lung tissue sample into a mortar precooled by liquid nitrogen, and add liquid nitrogen to fully grind it into powder. Samples in both groups were sonicated with four volumes of lysis buffer (8 M urea, 1% protease inhibitor, and 1% phosphatase inhibitor). Cell debris was removed by centrifugation at 12000 G for 10 min at 4 °C, and the supernatant was transferred to a new centrifuge tube for protein concentration determination by BCA kit.

### Pancreatin enzymolysis

Take the same amount of protein from the lung tissue of the two groups for enzymolysis, and adjust to the same volume with the lysis solution. Slowly add TCA to a final concentration of 20%, vortex and mix, and precipitate at 4 °C for 2 hours. Then apply 4500 g, centrifuge for 5 min, discard the supernatant, and wash the precipitate 2-3 times with precooled acetone. After the precipitate was air-dried, TEAB with a final concentration of 200 mM was added, the precipitate was broken up by ultrasound, and trypsin was added at a ratio of 1:50 (protease: protein, m/m) for enzymolysis overnight. Dithiothreitol (DTT) was added to a final concentration of 5 mM and reduced at 56 °C for 30 min. Then iodoacetamide (IAA) was added to a final concentration of 11 mM and incubated at room temperature for 15 min in the dark.

### Modification and enrichment

Dissolve the obtained peptide in the enrichment buffer solution (50% acetonitrile/6% trifluoroacetic acid), transfer the supernatant to the IMAC material washed in advance, and place it on a rotary shaker for incubation with mild shaking. After completion of the incubation, the resin was washed three times sequentially with the buffer solutions 50% acetonitrile/6% trifluoroacetic acid and 30% acetonitrile/0.1% trifluoroacetic acid. Finally, the modified peptide was eluted with 10% ammonia, and the eluate was collected and vacuum-frozen. Desalting was performed according to the instructions of C18 ZipTips after pumping, and the samples were subjected to liquid chromatography-mass spectrometry after vacuum freezing and pumping.

### Liquid chromatography-mass spectrometry

Peptides were dissolved in the mobile phase A of liquid chromatography and separated by NanoElute UPLC (Ultra Performance Liquid Chromatography, UPLC) system. Mobile phase A was an aqueous solution containing 0.1% formic acid and 2% acetonitrile, and mobile phase B was a solution containing 0.1% formic acid and 100% acetonitrile. Liquid gradient setting: 0-78 min, 2% -22% B; 78-84 min, 22% -35% B; 84-87 min, 35% -90% B; 87-90 min, 90% B, flow rate maintained at 450 nL/min. Peptides were separated by UPLC and injected into Capillary ion source for ionization and then analyzed by timsTOF Pro mass spectrometry. The ion source voltage was set to 1.7 kV, and both the peptide parent ion and its secondary fragments were detected and analyzed using high-resolution TOFs. The scanning range of the secondary mass spectrum is set to be 100-1700. Data acquisition was performed using the Parallel Accumulation Serial Fragmentation (PASEF) mode. After a primary mass spectrum is collected, the secondary spectrum with the charge number of the parent ion in the range of 0-5 is collected in PASEF mode for 10 times, and the dynamic exclusion time of tandem mass spectrum scanning is set to 30 s to avoid repeated scanning of the parent ion.

## Bioinformatics analysis methods

### Annotation methods

#### GO annotation

The Gene Ontology, or GO, is a major bioinformatics initiative to unify the representation of gene and gene product attributes across all species. More specifically, the project aims to: 1. Maintain and develop its controlled vocabulary of gene and gene product attributes; 2. Annotate genes and gene products, and assimilate and disseminate annotation data; 3. Provide tools for easy access to all aspects of the data provided by the project. The ontology covers three domains: 1. Cellular component: A cellular component is just that, a component of a cell, but with the proviso that it is part of some larger object; this may be an anatomical structure (e.g. rough endoplasmic reticulum or nucleus) or a gene product group (e.g. ribosome, proteasome or a protein dimer). 2. Molecular function: Molecular function describes activities, such as catalytic or binding activities, that occur at the molecular level. GO molecular function terms represent activities rather than the entities (molecules or complexes) that perform the actions, and do not specify where or when, or in what context, the action takes place. 3. Biological process: A biological process is series of events accomplished by one or more ordered assemblies of molecular functions. It can be difficult to distinguish between a biological process and a molecular function, but the general rule is that a process must have more than one distinct steps. GO annotation proteome was derived from the UniProt-GOA database (http://www.ebi.ac.uk/GOA/). Firstly, Converting identified protein ID to UniProt ID and then mapping to GO IDs by protein ID. If some identified proteins were not annotated by UniProt-GOA database, the InterProScan soft would be used to annotated protein’s GO functional based on protein sequence alignment method. Then proteins were classified by GO annotation based on three categories: biological process, cellular component and molecular function.

#### Domain annotation

A protein domain is a conserved part of a given protein sequence and structure that can evolve, function and exist independently of the rest of the protein chain. Each domain forms a compact three-dimensional structure and often can be independently stable and folded. Many proteins consist of several structural domains. One domain may appear in a variety of differentially expressed proteins. Molecular evolution uses domains as building blocks and these may be recombined in different arrangements to create proteins with different functions. Domains vary in length from between about 25 amino acids up to 500 amino acids in length. The shortest domains such as zinc fingers are stabilized by metal ions or disulfide bridges. Domains often form functional units, such as the calcium-binding EF hand domain of calmodulin. Because they are independently stable, domains can be “swapped” by genetic engineering between one protein and another to make chimeric proteins.Identified proteins domain functional description were annotated by InterProScan (a sequence analysis application) based on protein sequence alignment method, and the InterPro domain database was used. InterPro (http://www.ebi.ac.uk/interpro/) is a database that integrates diverse information about protein families, domains and functional sites, and makes it freely available to the public *via* Web-based interfaces and services. Central to the database are diagnostic models, known as signatures, against which protein sequences can be searched to determine their potential function. InterPro has utility in the large-scale analysis of whole genomes and meta-genomes, as well as in characterizing individual protein sequences.

#### KEGG pathway annotation

KEGG connects known information on molecular interaction networks, such as pathways and complexes (the “Pathway” database), information about genes and proteins generated by genome projects (including the gene database) and information about biochemical compounds and reactions (including compound and reaction databases). These databases are different networks, known as the “protein network”, and the “chemical universe” respectively. There are efforts in progress to add to the knowledge of KEGG, including information regarding ortholog clusters in the KEGG Orthology database. KEGG Pathways mainly including: Metabolism, Genetic Information Processing, Environmental Information Processing, Cellular Processes, Rat Diseases, Drug development. Kyoto Encyclopedia of Genes and Genomes (KEGG) database was used to annotate protein pathway. Firstly, using KEGG online service tools KAAS to annotated protein’s KEGG database description. Then mapping the annotation result on the KEGG pathway database using KEGG online service tools KEGG mapper.

#### Subcellular localization

The cells of eukaryotic organisms are elaborately subdivided into functionally distinct membrane bound compartments. Some major constituents of eukaryotic cells are: extracellular space, cytoplasm, nucleus, mitochondria, Golgi apparatus, endoplasmic reticulum (ER), peroxisome, vacuoles, cytoskeleton, nucleoplasm, nucleolus, nuclear matrix and ribosomes. Bacteria also have subcellular localizations that can be separated when the cell is fractionated. The most common localizations referred to include the cytoplasm, the cytoplasmic membrane (also referred to as the inner membrane in Gram-negative bacteria), the cell wall (which is usually thicker in Gram-positive bacteria) and the extracellular environment. Most Gram-negative bacteria also contain an outer membrane and periplasmic space. Unlike eukaryotes, most bacteria contain no membrane-bound organelles, however there are some exceptions. There, we used wolfpsort a subcellular localization predication soft to predict subcellular localization. Wolfpsort is an updated version of PSORT/PSORT II for the prediction of eukaryotic sequences. Special for protokaryon species, Subcellular localization prediction soft CELLO was used.

#### Functional enrichment

##### Enrichment of gene ontology analysis

Proteins were classified by GO annotation into three categories: biological process, cellular compartment and molecular function. For each category, a two-tailed Fisher’s exact test was employed to test the enrichment of the differentially expressed protein against all identified proteins. The GO with a corrected p-value < 0.05 is considered significant. Enrichment of pathway analysis: Encyclopedia of Genes and Genomes (KEGG) database was used to identify enriched pathways by a two-tailed Fisher’s exact test to test the enrichment of the differentially expressed protein against all identified proteins. The pathway with a corrected p-value < 0.05 was considered significant. These pathways were classified into hierarchical categories according to the KEGG website. Enrichment of protein domain analysis: For each category proteins, InterPro (a resource that provides functional analysis of protein sequences by classifying them into families and predicting the presence of domains and important sites) database was researched and a two-tailed Fisher’s exact test was employed to test the enrichment of the differentially expressed protein against all identified proteins. Protein domains with a corrected p-value < 0.05 were considered significant.

##### Enrichment-based clustering

For further hierarchical clustering based on differentially expressed protein functional classification (such as: GO, Domain, Pathway, Complex). We first collated all the categories obtained after enrichment along with their P values, and then filtered for those categories which were at least enriched in one of the clusters with P value <0.05. This filtered P value matrix was transformed by the function x = −log10 (P value). Finally these x values were z-transformed for each functional category. These z scores were then clustered by one-way hierarchical clustering (Euclidean distance, average linkage clustering) in Genesis. Cluster membership were visualized by a heat map using the “heatmap.2” function from the “gplots” R-package.

##### Protein-protein interaction network

All differentially expressed protein database accession or sequence were searched against the STRING database version 11.0 for protein-protein interactions. Only interactions between the proteins belonging to the searched data set were selected, thereby excluding external candidates. STRING defines a metric called “confidence score” to define interaction confidence; we fetched all interactions that had a confidence score ≥ 0.7 (high confidence). Interaction network form STRING was visualized in R package “networkD3”.

## Results

### Intraperitoneal injection of cecal contents to establish juvenile septic rat

About 4 hours after intraperitoneal injection of cecal content solution, 4-week-old SD rats have poor mental state, erect hair, accelerated breathing, curled up body, decreased vitality, reduced intake of water and slow response to stimulation, suggesting that pediatric rats have clinical manifestations of sepsis. Fifteen 4-week-old SD rats were intraperitoneally injected with cecal contents. 9 rats died within the observation time (72 hours), and the mortality was 60%. (**Supplementary Figure 1A**). Compared with the normal group, leukocytes, neutrophils and lymphocytes of rats with sepsis symptoms after intraperitoneal injection of cecal contents decreased significantly (P < 0.05), began to increase at 24 hours (P < 0.05), and monocytes decreased when pediatric rats had sepsis symptoms (P < 0.05), and began to rise at 9 hours. Platelets remained lower than normal level after the symptoms of sepsis in pediatric rats until the end of the observation period (P < 0.05) (**Supplementary Figure 1B**). Compared with the normal group, the expression of CRP, SAA, E-selectin, VEGFA, ICAM1 and NGAL at each time point after intraperitoneal injection of cecal content increased significantly (P < 0.05).and PGE2 decreased (P < 0.05) (**Supplementary Figure 1C**). After sepsis symptoms occurred in juvenile rats after intraperitoneal injection of cecal contents, HE staining of lung tissue showed thickening of alveolar wall, accompanied by granulocyte infiltration and falling cell fragments in local bronchus. 24 hours after the onset of sepsis symptoms occurred, HE staining of liver tissue showed that granular degeneration, loose cytoplasm and light staining of hepatocytes were widely seen around the central vein and portal area and in the liver parenchyma (**Supplementary Figure 1D**).

### Culture, amplification and identification of HUMSCs

When human umbilical cord tissue mass was incubated in mesenchymal stem cell culture medium for 5-7 days, it can be seen that some tissue mass adhered to the wall and became round, and new cells crawled out around it, showing spindle shape and sparse distribution (**Supplementary Figure 2A**). After incubating for 7-10 days, the density of primary cells increased obviously, and tended to fuse. The cells were slender and enlarged in size, similar to fibroblasts. The growth rate of primary cells is slow, about 10-14 days, and the fusion degree of cells reaches 80%-90%, which can be passed. After passage, the cells grew rapidly, with 1: 3 passage. In this experiment, the third generation cells were used. At this stage, the cells were long spindle-shaped and had good growth vitality (**Supplementary Figure 2B**). Eight surface markers of MSCs were selected as the surface markers for detecting HUMSCs, which were CD 73, CD 90, CD 105, CD 11B, CD 34, CD 45 and HLA-DR. In this experiment, the surface markers of MSCs were selected from the third generation cells. The results showed that HUMACs stably expressed CD73 (99.01%), CD90 (95.6%) and CD105 (96.2%), and expressed CD11B (0.80%), CD19 (0.6%), CD34 (0.4%), CD45 (0.6%) and HLA-DR (0.6%) (**Supplementary Figure 2C**).

### Proteomics dimension analysis of the mechanism of HUMSCs in the treatment of lung injury in juvenile septic rat

#### Difference of protein expression in lung tissue of juvenile septic rats treated with HUMSCs

**Figure 1:**
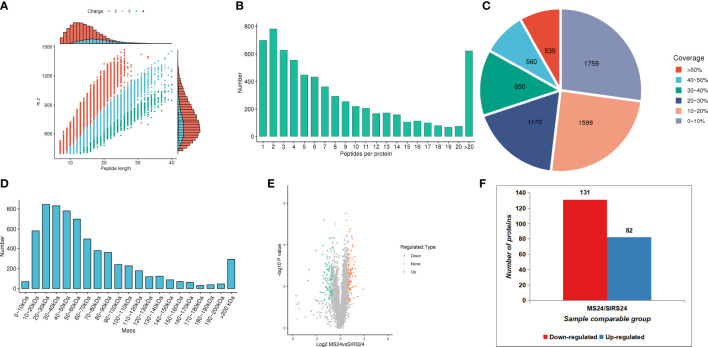
Proteomic quality control and DEPS identification after HUMSCs intervention. Peptide length distribution: most of the peptides were distributed in 7-20 amino acids **(A)**. Peptide number distribution: most proteins corresponded to more than two peptides **(B)**. Protein coverage distribution: the coverage of most proteins is below 30% **(C)**. Protein molecular weight distribution: the molecular weight of the identified protein is present at different stages and is evenly distributed **(D)**. Volcanogram showed the difference of protein expression between MS24 and SIRs24 group in lung tissue of juvenile septic rats **(E)**. The DEPS comparison between MS24 and SIRs24. 213 proteins were identified as DEPS (3.3% of 6438 proteins), of which 82 proteins were up-regulated and 131 proteins were down-regulated **(F)**.

In order to fully understand the mechanism of lung injury repair in juvenile septic rats after HUMSCs intervention, quantitative information was obtained by label-free quantitative proteomics. Based on the results of database search, the quality control analysis of peptides and modification sites was carried out on the lung tissues of juvenile septic rats 24 hours after HUMSCs intervention (MS24) and the lung tissues of juvenile septic rats without intervention (SIRs24). Most of the peptides were distributed in 7-20 amino acids, which was consistent with the general rules based on enzymatic digestion and mass spectrometry fragmentation. The distribution of peptide lengths identified by mass spectrometry met quality control requirements (**Figure 1A**); most proteins corresponded to more than two peptides. During quantification, one protein corresponding to multiple specific peptides (or corresponding to multiple spectra) is conducive to increasing the accuracy and credibility of the quantification results (**Figure 1B**); the coverage of most proteins is below 30% (**Figure 1C**); the molecular weight of the identified protein is present at different stages and is evenly distributed (**Figure 1D**). The peptide length distribution, peptide number distribution, protein coverage distribution and protein molecular weight distribution all meet the quality control requirements. Volcanogram showed the difference of protein expression between MS24 and SIRs24 group in lung tissue of juvenile septic rats(**Figure 1E**). A total of 213 proteins (3.3% of 6438 proteins) were identified as difference of protein expression (DEPS) after HUMSCs intervention, of which 82 proteins were up-regulated and 131 proteins were down-regulated (**Figure 1F**), the top 20 proteins up-regulated and the top 20 proteins down-regulated (**Supplementary Table 2** **)**.

#### Main functional protein mechanism of HUMSCs in treating lung injury in juvenile septic rats

##### GO annotation classification

**Figure 2:**
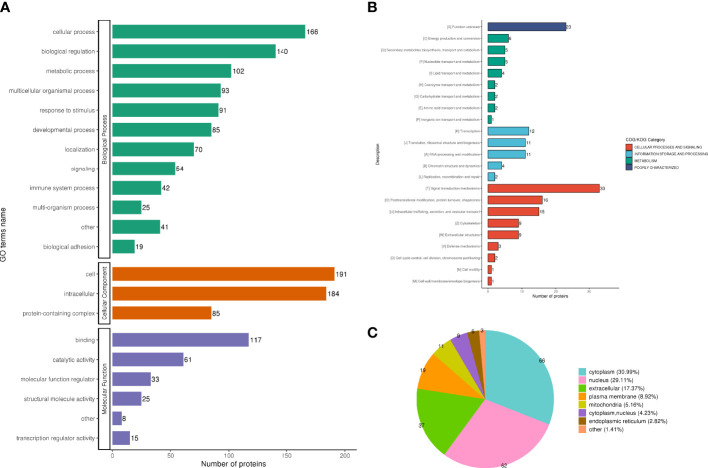
Functional classification of DEPS. GO classification, in the biological process classification, most proteins are involved in cellular processes and biological regulation; in the cellular component classification, most proteins are distributed in intracellular fluid and protein-containing complexes; in the molecular function classification, most of the proteins are in the categories of binding, catalytic activity and molecular regulators **(A)**. The subcellular structure was dominated by cytoplasm and nucleus **(B)**. COG functional classification showed that most of the proteins were in the signal transduction machinery category **(C)**.

The analysis according to GO classification (**Figure 2A**) shows that in the classification of biological processes, most proteins are involved in cellular processes and biological regulation; cellular components are dominated by intracellular fluid and protein-containing complexes; and molecular functions are classified in the categories of binding, catalytic activity, and molecular regulators. The immune system process categories contained 42 DEPSs, Mx1 (ratio 4.5, up-regulated), Rap2c (ratio 2.9481, up-regulated), TNC (ratio 2.8903, up-regulated), Arrdc1 (ratio 2.8117, up-regulated), AIF1 (ratio 0.4886, Down-regulation), MIF (ratio 0.4862, down-regulation) and UBXN1 (ratio 0.4776, down-regulation) were involved in the process of immune inflammation.

##### Subcellular structure annotation classification

In the subcellular structure annotation classification, the subcellular structure is dominated by cytoplasm (30.99%) and nucleus (29.11%) (**Figure 2B**).

##### COG functional classification

In the COG functional classification (**Figure 2C**), most of the proteins are in the signal transduction mechanism category (including 33 DEPS).

#### Main protein pathway mechanism and protein interaction network of HUMSCs in the treatment of lung injury in juvenile septic rats

##### Enrichment analysis of GO, KEGG pathways and protein domains of DEPs

GO classification, KEGG pathway and protein domain were used to analyze the differentially expressed proteins in the two groups.

**Figure 3:**
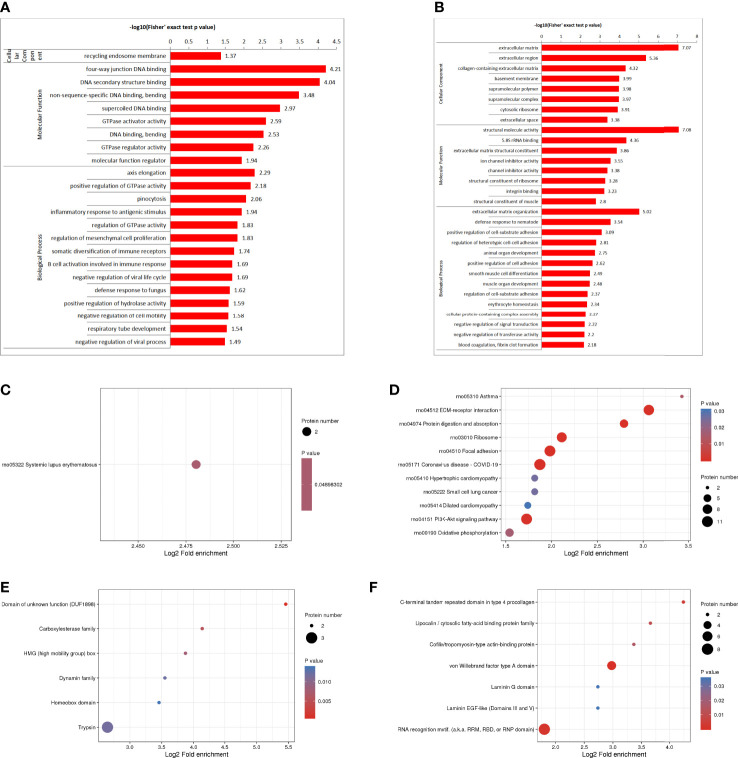
DEPS enrichment analysis. GO enrichment, up-regulated DEPS enrichmen **(A)**. GO enrichment, down-regulated protein enrichment **(B)**. KEGG enrichment of up-regulated DEPS **(C)**. KEGG enrichment of down-regulated DEPS **(D)**. Enrichment of important protein domains in upregulated DEPS **(E)**. Enrichment of important protein domains in downregulated DEPs **(F)**.

The GO enrichment analysis showed (**Figures 3A, B**), based on the up-regulated protein enrichment in the biological process, the positive regulation of GTPase activity, pinocytosis, antigen-stimulated inflammatory response, regulation of GTPase activity, regulation of mesenchymal stem cell proliferation, and regulation of B cell activation involved in immune response were enriched after the intervention of HUMSCs. The enrichment of down-regulated proteins in biological processes, including extracellular matrix organization, positive regulation of cell-matrix adhesion, positive regulation of cell-matrix adhesion, negative regulation of signal transduction, negative regulation of transferase activity, blood coagulation, fibrin clot formation, etc. Enrichment of up-regulated proteins based on molecular functions, such as class four-way junction DNA binding, DNA secondary structure binding, non-sequence-specific DNA binding, bending supercoiled DNA binding, GTPase activator activity, DNA binding and bending GTPase regulatory activity, molecular function regulators were enriched; Down-regulated proteins including structural molecule activity, 5.8 S rRNA binding, extracellular matrix structural components, ion channel inhibitor activity, channel inhibitor activity, structural components of ribosomes, and integrin binding were significantly present. In the classification of cell components, the up-regulated proteins were mainly enriched in the circulating endoplast membrane, and the down-regulated proteins were enriched in the extracellular matrix, extracellular zone, collagen-containing extracellular matrix, basement membrane, supramolecular polymers, supramolecular complexes, cytoplasmic ribosomes, extracellular space and other categories.

For KEGG enrichment of DEPS, we identified only 1 pathway from upregulated DEPS (**Figure 3C**) and 11 pathways from downregulated DEPs (**Figure 3D**). All of the significantly enriched pathways, ECM receptor interaction, PI3K-Akt signaling, and oxidative phosphorylation appear to be involved in the regulation of inflammatory responses.

Protein domain enrichment analysis, finding enrichment of important protein domains in upregulated DEPs, such as carboxylesterase family, HMG (High Mobility Group) box, trypsin, Dynamin family, homeobox domain (**Figure 3E**); The enrichment of important protein domains in down-regulated DEPs, It mainly includes von Willebrand factor type a domain, RNA recognition motif, C-terminal tandem repeat domain of type 4 procollagen, Lipocalin/cytoplasmic fatty acid-binding protein family, Cofilin/tropomyosin-type actin-binding protein, laminin-EGF-like domain (III and V), Laminin G domain (**Figure 3F**).

##### Cluster analysis of differentially expressed proteins

**Figure 4:**
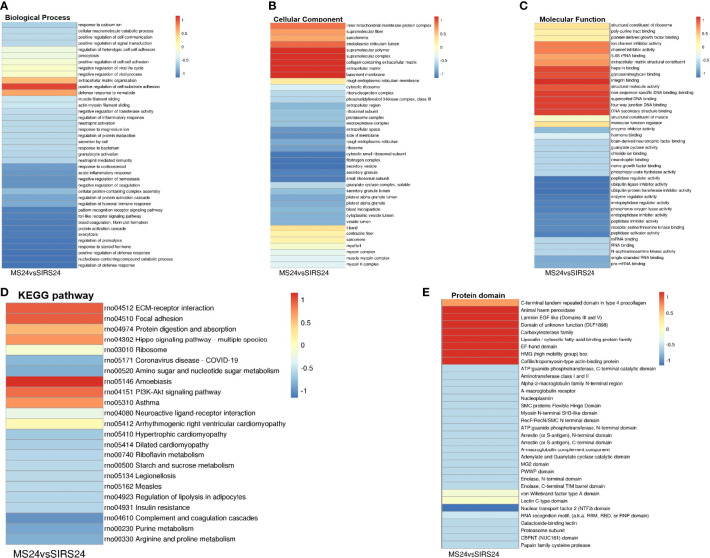
Cluster analysis of DEPS. The biological process cluster analysis of GO **(A)**. The cellular component analysis of GO **(B)**. The molecular function analysis **(C)**. The KEGG pathway cluster analysis **(D)**. Tthe protein domain cluster analysis **(E)**.

Go cluster analysis showed that DEPS was related to the positive regulation of cell-matrix adhesion, and the main cellular components were collagen-containing extracellular matrix, extracellular matrix and basement membrane. KEGG pathway cluster analysis showed that most proteins were related to ECM receptor interaction and PI3K-Akt signaling pathway; Cluster analysis of protein domains revealed a predominance of EFG-like laminin (**Figure 4**).

##### PPI of differentially expressed proteins

PPI networks can identify and characterize relevant protein complexes, which is essential for understanding the molecular events involved. In order to clearly show the interaction between proteins, we selected the top 50 proteins with the closest interaction, and selected the top 15 proteins as hub proteins according to the degree (**Supplementary Table 3**). We found that some proteins were involved in the regulation of immune inflammatory response.

**Figure 5:**
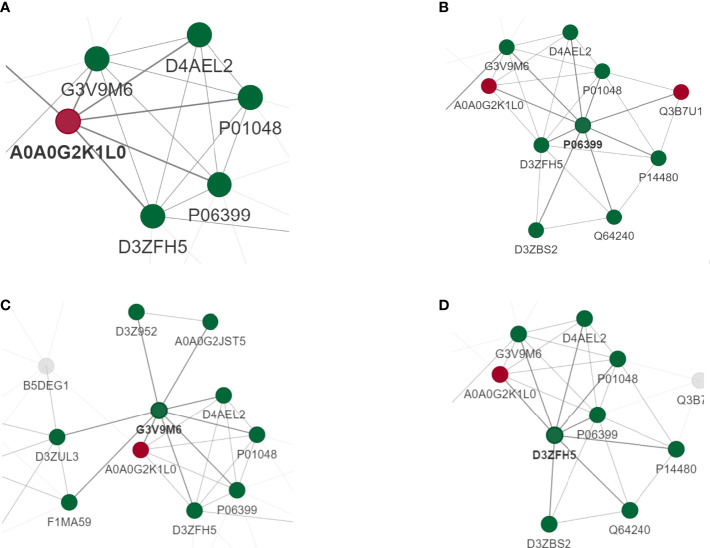
PPI analysis of DEPS. MCODE obtained 36 modules and extracted 1 up-regulated DEP module **(A)** and 3 most significant down-regulated DEP modules **(B–D)**.

To perform PPI topology analysis, we combined known biomarker DEPs and applied them to plug-ins in the Molecular Complex Detection (MCODE) cellular landscape. Separation of dense regions and prediction of protein complexes by PPI subnetworks. MCODE got 36 modules and extracted 1 up-regulated DEP module (**Figure 5A**) and 3 most significant down-regulated DEP modules (**Figures 5B–D**).

#### Mechanism of HUMSCs in the treatment of lung injury in juvenile septic rats by phosphorylation modification omics dimension analysis

##### Difference of protein phosphorylation modification of HUMSCs in the treatment of lung injury in juvenile septic rats

**Figure 6:**
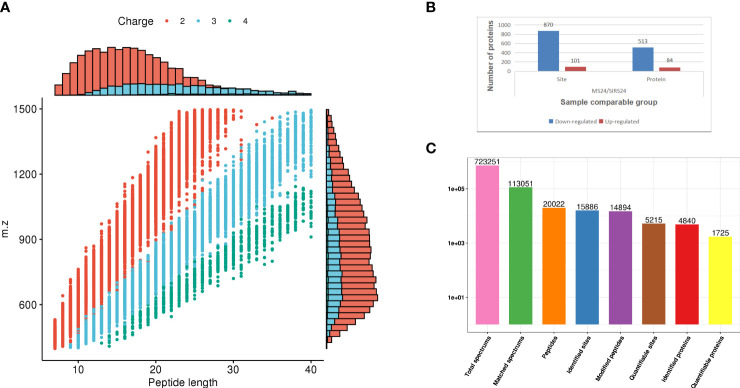
Data quality control and quantitative analysis of phosphorylation sites. The distribution of peptide length identified by mass spectrometry meets the quality control requirements **(A)**. 4840 phosphorylation sites were identified by protein phosphorylation analysis, of which 1725 were quantified **(B)**. 971 phosphorylation sites were significantly changed among all the quantified phosphorylation sites, of which 101 sites were up-regulated and 870 sites were down-regulated **(C)**.

Quality control evaluation is required for the data of mass spectrometry after the database search is completed. Most of the peptides are distributed in 7-20 amino acids, which is in line with the general law based on enzymolysis and mass spectrometry fragmentation. The distribution of peptide lengths identified by mass spectrometry met the quality control requirements (**Figure 6A**). Phosphorylation changes in lung injury of juvenile septic rats after HUMSCs intervention were analyzed by protein phosphoromics, and 4840 phosphorylation sites were identified, of which 1725 sites were quantified (**Figure 6B**). Among all the quantified phosphorylation sites, 971 phosphorylation sites were significantly changed, of which 101 sites were up-regulated and 870 sites were down-regulated (**Figure 6C**).

### Functional mechanism of DDPs in the treatment of lung injury in juvenile septic rats with HUMSCs

To investigate the protein phosphorylation modification group in the lung tissue of juvenile septic rats after HUMSCs intervention, we classified the phosphorylated proteins by GO, subcellular localization, and COG.

#### GO annotation classification

**Figure 7:**
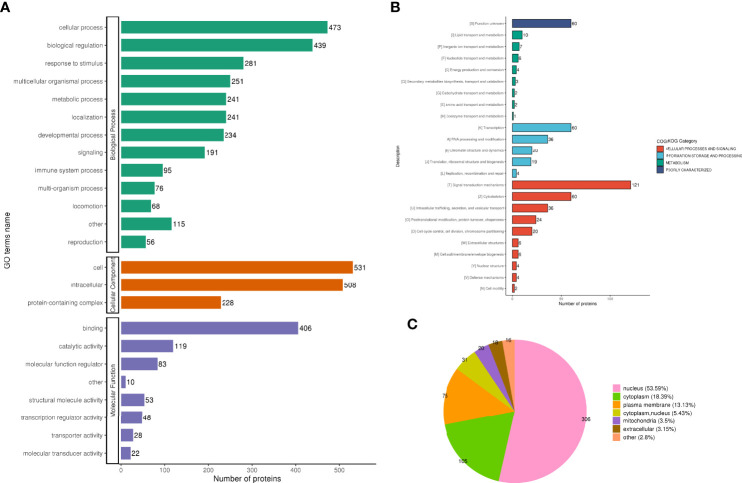
Functional classification of DPPs. GO classification analysis, the two biological processes with the greatest changes in DPPs are cellular progression and biological regulation, the cellular components are mainly cells and intracellular fluids, and the molecular functions are mainly protein binding and catalytic activity **(A)**. subcellular localization analysis, 571 phosphorylated proteins were quantitatively distributed in the nucleus (53.59%), cytoplasm (18.39%), and plasma membrane (13.13%) **(B)**. COG functional classification, most of the phosphorylated proteins that can be quantified are mainly in the categories of signal transduction mechanism (121 DPPs), transcription (60 DPPs), and cytoskeleton (60 Dpps) **(C)**.

The two biological processes with the greatest changes in DPPs are cellular progression and biological regulation, the cellular components are mainly cells and intracellular fluids, and the molecular functions are mainly protein binding and catalytic activity (**Figure 7A**); The immune system process categories contained 95 DPPs, among which Rps17 (ratio 3.4465, up-regulated), ifitm3 (ratio 2.1523, up-regulated), Psme2 (ratio 2.2593, up-regulate), Snap29 (ratio 2.1905, up-regulate), Dync1i2 (ratio 0.2828, Down), Cd93 (ratio 0.2958, down), Cav1 (ratio 0.173, down), Pik3r4 (ratio 0.3905, down Down-regulation) and Lrrc8a (ratio 0.1174, down-regulation) were involved in the process of immune inflammation.

#### Subcellular localization analysis 

Five hundred and seventy-one phosphorylated proteins were quantitatively distributed in the nucleus (53.59%), cytoplasm (18.39%), and plasma membrane (13.13%) (**Figure 7B**).

#### Functional classification of COG

Most of the phosphorylated proteins that can be quantified are mainly in the categories of signal transduction mechanism (containing 121 DPPs), transcription (containing 60 DPPs), and cytoskeleton (containing 60 Dpps) (**Figure 7C**).

#### Key differential phosphorylated protein pathway mechanism and protein interaction network of HUMSCs in the treatment of lung injury in juvenile septic rats

##### GO and KEGG pathway enrichment analysis of DPPS

###### GO enrichment analysis of DPPs (**Figures 8A, B**)

In the HUMSCs intervention group, according to the classification of biological process enrichment, the response to rapamycin, plasma membrane repair, assembly of protein-containing complexes, oxidative phosphorylation, cortical cytoskeleton organization, RNA metabolic process, striated muscle contraction, and modification of synaptic structure were all enriched in the up-regulated phosphorylated proteins. In contrast, the regulation of cytoskeletal organization, organelle organization, supramolecular fiber organization, actin cytoskeletal organization, organelle organization and cell-substrate junction assembly is largely enriched in down-regulated phosphorylated proteins. Molecular function enrichment analysis showed that the binding categories were significantly enriched, such as RNA binding, complex-containing binding proteins, mRNA binding, actin filament binding, actin binding and structural molecular activity, which were enriched in up-regulated phosphorylated proteins. The down-regulated phosphorylated proteins mainly include structural components of the cytoskeleton, structural molecular activity, cytoskeletal protein binding, actin filament binding, and intercellular adhesion mediator activity. In cell component enrichment classification analysis, highly enriched categories included protein-containing complexes of upregulated phosphorylated proteins, ribonucleoprotein complexes, actin-based cell projection clusters, erect cilia, spliceosome complexes, erect cilia bundles, plasma membrane regions and cell division sites. For down-regulated phosphorylated proteins, cell junctions, adherens junctions, anchor junctions, intercellular junctions, and plasma membrane regions were largely enriched.

**Figure 8:**
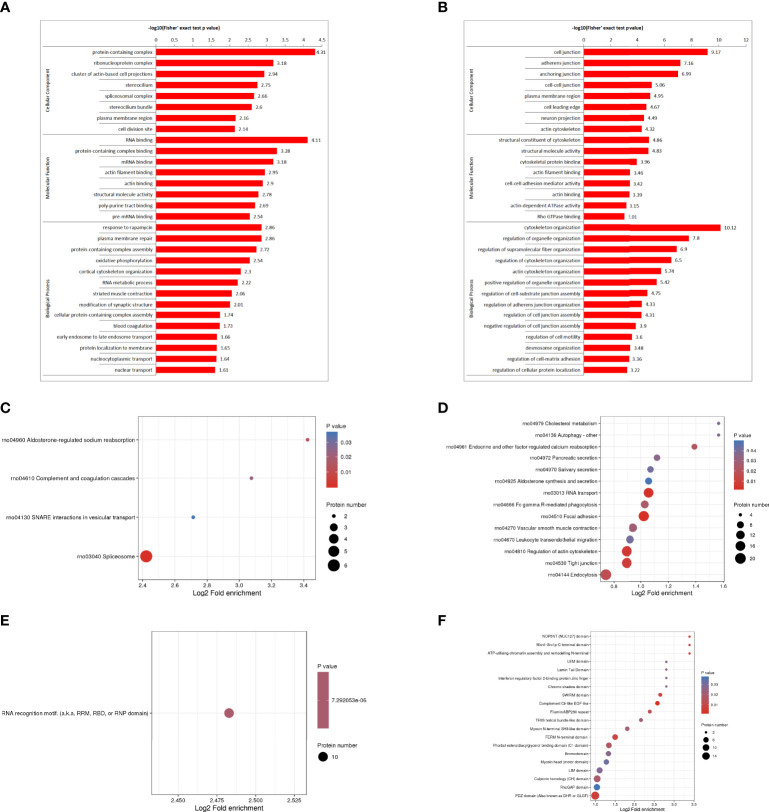
Functional enrichment analysis of DDPs. GO enrichment, up-regulated differentially phosphorylated protein enrichment **(A)**. GO enrichment, down-regulated differentially phosphorylated protein enrichment **(B)**. KEGG enrichment of up-regulated differently phosphorylated proteins **(C)**. KEGG enrichment of down-regulated differently phosphorylated proteins **(D)**. Enrichment of important protein domains in up-regulated differentially phospho-proteins **(E)**. Enrichment of important protein domains in down-regulated DDPs **(F)**.

###### KEGG pathway enrichment analysis of DPPs (**Figures 8C, D**)

KEGG pathway analysis was performed on DPPs, and 4 pathways were identified from the upregulated DPPs, including spliceosome, aldosterone-regulated sodium reabsorption, complement and coagulation cascade, and trap interaction pathway in vesicular transport. Phosphoproteome data showed that phosphorylation levels were down-regulated in focal adhesion pathway, RNA transport pathway, actin cytoskeleton regulation pathway, tight junction pathway, endocytosis pathway, endocrine and other factors regulating calcium reabsorption pathway and FcγR-mediated phagocytosis pathway. Tight junctions encode genes for epithelial intercellular connexins, indicating that there is interference in the permeability between epithelial barriers, which may be closely related to the regulation of intercellular barriers in septic lung injury (**Supplementary Figure 4**).

###### Protein domain enrichment analysis of DPPs

We found that only RNA recognition motifs (RNP domains) were enriched in upregulated DPPs (**Figure 8E**). Enrichment of important protein domains in down-regulated DPPs, mainly including complement CLR-like EGF-like, PDZ domain, SWIRM domain, FERM N-terminal domain, NOP5NT (NUC127) domain, Man1-Src1p-C-terminal domain, ATP-utilizing chromatin assembly, and N-terminal remodeling (**Figure 8F**).

##### Cluster analysis and protein interaction network (PPI) of DDPs

**Figure 9:**
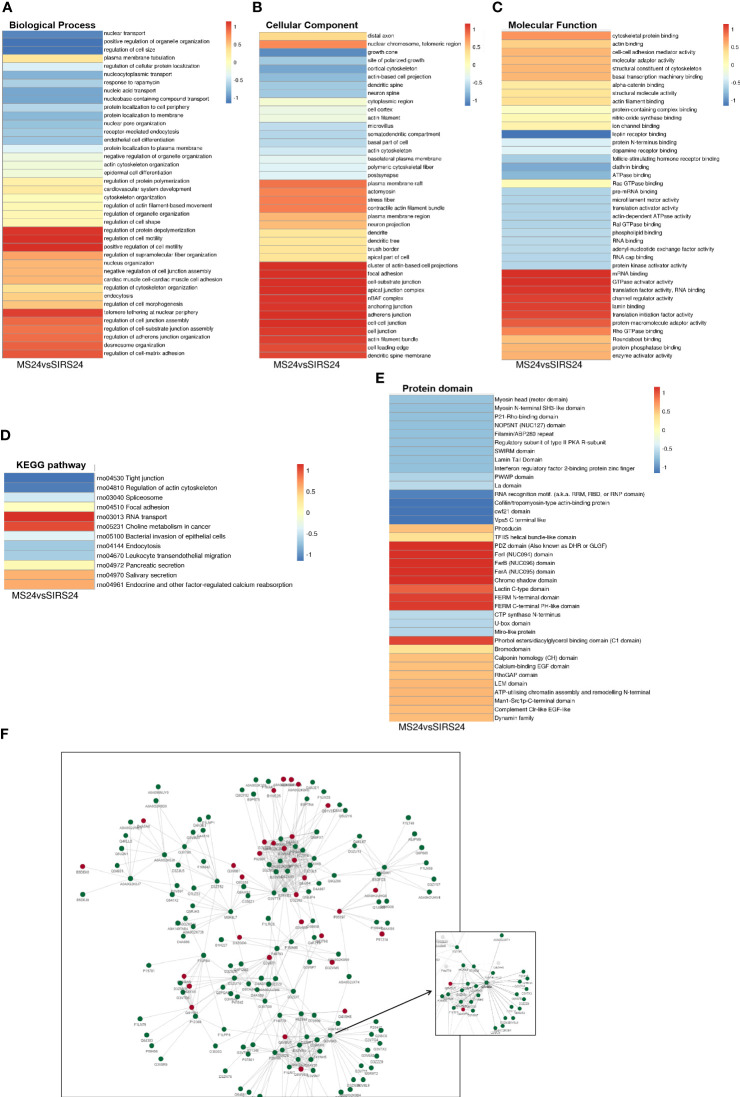
Cluster Analysis and Protein interaction network (PPI) of DDPs. Biological process cluster analysis of GO **(A)**. Cell component analysis of GO **(B)**. Molecular function analysis **(C)**. Cluster analysis of KEGG pathway **(D)**. Cluster analysis of protein domains **(E)**. The top 50 phosphorylated proteins with the closest interaction were selected, and a network containing a total of 173 related phosphorylated proteins was generated. G3V6K6 (EGFR) has 29 nodes **(F)**.

Go cluster analysis showed that DPPs were associated with the regulation of protein unwinding and polymerization, the regulation of cell motility, and the positive regulation of cell motility (**Figure 9A**). Cellular components are dominated by actin-based cell projection clusters, focal adhesions, cell-base junctions, apical junction complexes, nBAF complexes, anchored junctions, adherens junctions, intercellular junctions, etc. (**Figure 9B**). Molecular function analysis showed that most of the phosphorylated proteins were associated with mRNA binding, GTPase activating factor activity, translation factor activity, RNA banding, channel regulator activity, etc. (**Figure 9C**). Cluster analysis of the KEGG pathway revealed that most of the phosphorylated proteins were involved in RNA transport (**Figure 9D**). Cluster analysis of the protein structural domains showed that they were closely related to the PDZ domain, FerI domain, FerB domain, FerA domain, chromosome shadow domain, etc. (**Figure 9E**).

We selected the top 50 phosphorylated proteins with the closest interaction and generated a network containing a total of 173 related phosphorylated proteins (**Figure 9F**). The Phosphorylated proteins with more interactions include Egfr, Sf3b1, U2af2, Srsf1, Srrm1, Arrb1, Hnrnpd, Igf2r, Srsf11 and Srrm2. Among them, G3V6K6 (EGFR) has 29 nodes, D4A9L2 (SRSF1) has 24 nodes, Q9JJ54 (Hnrnpd) has 21 nodes and G3V824 (Igf2r) has 21 nodes. It is also valuable for other analyses in this study. G3V6K6 (EGFR) interaction is the most (29 nodes), which may be involved in the repair process of septic lung injury.

##### Motif analysis of key phosphorylation sites of HUMSCs in the treatment of lung injury in juvenile septic rats

Protein phosphorylation is regulated by protein kinases (PKs), and different PKs prefer specific substrates with conserved motifs. We performed a bioinformatic analysis to identify novel phosphorylation motifs using a large number of phosphorylation sites found in this study. We performed intensive sequence analysis of hyperphosphorylated site motifs around the phosphate groups (10 amino acids upstream and 10 amino acids downstream of each phosphate group) of serine and threonine residues using the Motify-X program.

Among the 1316 phosphorylation sites, we identified 123 conserved motifs based on phosphorylated serine (pS) phosphorylation sites and 17 conserved motifs based on phosphorylated threonine (pT) phosphorylation sites.

**Figure 10:**
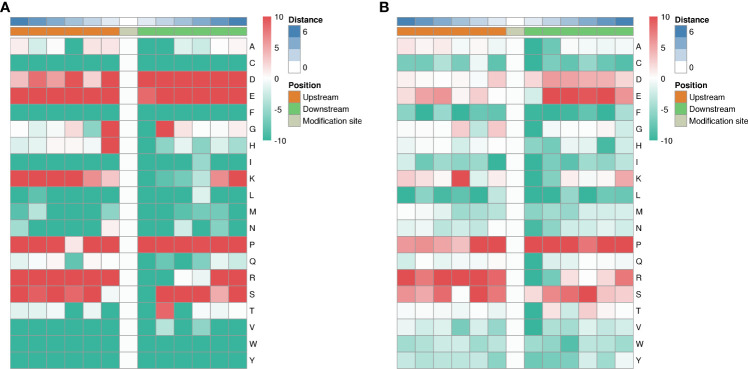
Motif analysis of phosphorylation sites. Heat map showing enrichment or depletion of specific amino acids near the serine phosphorylation site. D, E, P, R, and S tend to be near the serine phosphorylation site **(A)**. P, R, S are largely represented in the proximal region of the threonine phosphorylation site **(B)**.

We generated heat maps (**Figure 10A**) to show the enrichment or depletion of specific amino acids near the serine phosphorylation site. D, E, P, R and S have a tendency to move to the vicinity of serine phosphorylation sites. P, R, S are largely represented in the proximal region of the threonine phosphorylation site (**Figure 10B**). These amino acids close to the phosphorylation sites preferentially reflect the specific recognition of enzymes that catalyze phosphorylation in septic lung tissue after HUMSCs intervention. Further studies are needed to investigate whether different types of enzymes and kinases are active in regulating phosphorylation.

## Discussion

Sepsis is a systemic inflammatory response syndrome caused by external pathogenic microorganism infection. The development of children’s immune system is still imperfect, which can easily lead to multiple organ failure. The most common complications of ALI (20, 21), which can lead to bronchial epithelial cell injury, inflammatory cell infiltration in alveolar wall, and severe damage of lung blood barrier-capillary barrier. Cause respiratory failure (22). Studies have shown that MSC can alleviate ALI in sepsis through direct or paracrine effects (23, 24). In recent years, studies have confirmed that HUMSCs have immunomodulatory properties (4), and have made progress in the treatment of sepsis preclinical research (25).

The results of our study showed that the swelling of bronchial epithelial cells and the inflammatory cells on the alveolar wall were reduced by he staining after 24 hours of HUMSCs intervention (**Supplementary Figure 1**); the damage of lung blood and air barrier-capillary was reduced by transmission electron microscopy (**Supplementary Figure 4**). Endothelial cytokines E-selectin, VEGFA, ICAM1, and NGAL were significantly decreased, and PGE2 was elevated (**Supplemental Figure 5**). In conclusion, HUMSCs intervention can significantly reduce the inflammation of endothelial cells in blood circulation and lung tissue, improve the lung gas-blood barrier, and promote the repair of lung injury in juvenile septic rats. However, its molecular mechanism still needs to be further studied and clarified.

Phosphorylation modification is the most basic, common and important mechanism to regulate protein activity and function (11). Phosphorylation modification is the most basic, common and important mechanism to regulate protein activity and function (11). In order to comprehensively understand the mechanism of lung injury repair after HUMSCs intervention in juvenile septic rats, we performed proteomics and phosphoromics analysis of lung tissues of juvenile septic rats 24 hours after HUMSCs intervention (MS24) and those of unintervened septic juvenile rats (SIRs24) to further clarify its molecular mechanism.

First, we analyzed the mechanism of HUMSCs in the treatment of lung injury in juvenile septic rats from the perspective of proteomics. The results showed that 213 proteins were identified as differentially expressed proteins after HUMSCs intervention, of which 82 proteins were up-regulated and 131 proteins were down-regulated compared with the lung tissues of juvenile septic rats without intervention. It may be a potential key protein for the intervention of HUMSCs to alleviate septic lung injury.

According to GO classification analysis, in the classification of biological processes, most proteins are involved in cellular processes and biological regulation; they are mainly distributed in intracellular fluid and protein-containing complexes; their functions are mainly in the categories of binding and catalytic activity. It is worth noting that there are 42 DEPS in the immune system process category, among which the up-regulated differential proteins Mx1, Rap2c, TNC and Arrdc1 and the down-regulated differential proteins AIF1, MIF and UBXN1 are involved in the immune inflammatory response process. It has been reported that MX1 shows the key structural motif of GTPase family and is activated by rhabdovirus vaccination and bacterial RNA stimulation (26), and up-regulated with anti-inflammatory effect (27). Rap2c encodes a protein that is a member of the Ras-associated protein subfamily of the Ras-GTPase superfamily and acts as a molecular switch to regulate cell proliferation, differentiation, and apoptosis (28). TNC is an extracellular matrix (ECM) glycoprotein whose regulation is closely related to inflammation (29). Arrdc1 is involved in a variety of processes including cellular protein metabolism, extracellular vesicle biogenesis, and negative regulation of the Notch signaling pathway (30).The down-regulated differential protein AIF1 has been identified as an up-regulated protein of infection (31). The ubiquitin-regulated X (UbX) protein UbXN1, which contains a ubiquitin-associated domain, is a negative regulator of nuclear factor-κB (NF-κB) signaling (32, 33). It is suggested that the immune inflammatory response is important in the proteome of septic lung tissue. Our results showed that E-selectin, VEGFA, ICAM1 and NGAL were significantly decreased and PGE2 was increased after HUMSCs intervention (**Supplementary Figure 5**). It has been reported that TNC is an extracellular matrix (ECM) glycoprotein, and its upregulation can induce the expression of PGE2 (34), Macrophage migration inhibitory factor (MIF) is a multifunctional pro-inflammatory factor, and its overexpression can up-regulate the amount of ICAM-1, promote leukocyte adhesion to vascular endothelium, and mediate endothelial cell injury (35). After the intervention of HUMSCs, the content of ICAM-1 decreased and the content of PGE2 increased in juvenile septic rats, which participated in tissue repair, suggesting that HUMSCs may promote the repair of endothelial barrier injury in lung tissue by up-regulating the expression of TNC and PGE2, and (or) down-regulating the expression of MIF and ICAM-1. Studies have found that TNC is an ECM glycoprotein, and its regulation is closely related to inflammation. HUMSCs could significantly increase the level of PGE2 after intervention, while the up-regulation of TNC could induce the expression of PGE2. Therefore, we speculate that TNC may be the key protein for HUMSCs to promote the repair of lung injury in juvenile septic rats.

According to the results of go enrichment analysis, HUMSCs intervention participates in the regulation of antigen-stimulated inflammatory response and other biological processes, and is closely related to immune inflammatory response (36–38). For KEGG enrichment of differentially expressed proteins (DEPs), we identified only 1 pathway from upregulated DEPs and 11 pathways from downregulated DEPs. The main pathways related to immune inflammatory response are ECM receptor interaction pathway, focal adhesion pathway and other signaling pathways (39–41).

Protein domain refers to a specific component that appears repeatedly in different protein molecules, with similar sequence, structure and function, and is a conserved unit in the process of protein evolution (42). We found an enrichment of important protein domains, such as carboxylesterase family, HMG (high mobility group) box, in upregulated DEPs and an enrichment of important protein domains, mainly including von Willebrand factor type A domain, in downregulated DEPs. Studies have reported that the expression and activity of carboxylesterases (CEs) are down-regulated under inflammatory conditions (43). The high mobility group (HMG-Box) protein family is a class of nuclear proteins that are secreted from the nucleus to the outside of the cell during systemic inflammatory response and mediate the inflammatory response as a proinflammatory cytokine (44). Von Willebrand factor (von Willebrand factor, vWF) is a multi-domain synthesized by vascular endothelial cells and bone marrow megakaryocytes, which can be used as an important indicator of endothelial cell activation and endothelial dysfunction, and participate in the process of body inflammation, tissue injury and repair, and immune regulation (45). The results showed that CEs and HMG-Box were up-regulated and vWF domain was down-regulated after HUMSCs intervention, suggesting that HUMSCs played a regulatory role.

Cluster analysis showed that the DEPs were related to the positive regulation of cell-matrix adhesion.The KEGG pathway cluster analysis showed that most of the proteins were related to the ECM receptor interaction pathway and focal adhesion signaling pathway. The protein domain cluster analysis showed that EFG-like laminin was the main protein.

GO enrichment analysis showed that HUMSCs intervened in regulating GTPase activity and antigen-stimulated inflammatory response, and B cell activation participated in immune response, extracellular matrix tissue, cell-matrix adhesion and other biological processes, which were closely related to immune inflammatory response. The results of DEPs enrichment and cluster analysis suggested that HUMSCs may regulate the expression of CEs, HMG-Box and von Willebrand factor type a domains through ECM receptor interaction pathway signaling pathway, and regulate the immune inflammatory response in the lung tissue of juvenile septic rats.

PPI networks can identify and characterize relevant protein complexes, which is crucial for understanding the molecular events involved (46). We use the string to determine the relation for PPI (47). In order to clearly show the interaction between proteins, we selected the top 50 proteins with the closest interaction, and we selected the top 15 proteins as hub proteins according to the degree. These proteins, due to their high degree of interaction, separate dense regions and predict protein complexes through the PPI subnetwork. MCODE obtained 36 modules and found that one up-regulated DEP module was TNC and the three most significant down-regulated DEP modules were (Fga, Fbn1, Itih2). Therefore, we speculate that HUMSCs may activate TNC-mediated PGE2 release through ECM-receptor interaction pathway.

In addition, our study also analyzed the mechanism of HUMSCs intervention in reducing lung injury in juvenile septic rats from the perspective of protein phosphorylation. Protein phosphorylation plays an important role in the process of cell signal transduction and is the most common and important mechanism to regulate protein function (11). To investigate the phosphoproteome of lung tissue in juvenile septic rats after HUMSCs intervention, we performed GO, subcellular localization, and COG classification of phosphoproteins. The two biological processes with the greatest changes in DPPs are cellular progression and biological regulation, which are mainly distributed in intracellular fluid, and their molecular functions are mainly protein binding and catalytic activity. The up-regulated phosphorylated proteins ifitm3 and Snap29 and the down-regulated proteins Cd93, Mx1, Cmklr1, Adam17 and Lrrc8a are involved in the immune inflammatory response. Studies have reported that IFITM3 is involved in cell adhesion, apoptosis, immunity, and antiviral activity. In addition, the IFITM3 gene is considered to be a preferred marker for inflammatory diseases (48). SARS-CoV-2 infection hinders autophagic flux by upregulating GSK3B in lung cell lines or downregulating autophagic genes, SNAP29, and lysosomal acidification genes in human samples, resulting in increased viral replication, suggesting that SNAP29 upregulation after HUMSCs intervention contributes to the repair of lung tissue cells (49). DPPs such as Cd93, Mx1, Cmklr1, Adam17 and Lrrc8a were significantly down-regulated after HUMSCs intervention, suggesting that the repair of septic lung injury is also closely related to the down-regulation of Cd93, Mx1, Cmklr1, Adam17 and Lrrc8a after HUMSCs intervention (50–54). In this study, the down-regulated phosphorylated proteins were more obvious than the up-regulated ones. In the phosphoproteomic analysis, the above proteins may be potential phosphorylated proteins for intervention of HUMSCs to alleviate septic lung injury.

In order to better understand the function of the identified phosphorylated proteins, we performed enrichment analysis of DPPs in the two groups at three levels of GO classification, KEGG pathway, and protein domain, respectively. In the HUMSCs intervention group, regarding the enrichment of phosphorylated proteins based on biological processes, response to rapamycin (mTOR) was significantly enriched for up-regulated proteins and cytoskeletal organization was significantly enriched for down-regulated proteins. For the HUMSCs intervention group, phosphorylated proteins were enriched based on molecular function upregulation, such as RNA binding, complex-containing binding proteins, mRNA binding, etc. In cell component enrichment classification analysis, the highly enriched categories included protein-containing complexes of up-regulated phosphorylated proteins, ribonucleoprotein complexes, and for down-regulated phosphorylated proteins, cell junctions, adherens junctions, anchor knots, etc. Were significantly enriched.

Site occupancy of phosphorylation correlates with cell signaling status (55). KEGG pathway analysis was performed on DPPs to assess the pathways significantly represented in the intervention of HUMSCs in septic lung injury. We identified 4 pathways from upregulated DEPs and 14 pathways from downregulated DEPs. The down-regulated genes encoding epithelial intercellular connexin in the tight junction pathway and focal adhesion pathway indicate that there is interference in the permeability between epithelial barriers, which may be closely related to the regulation of intercellular barriers in septic lung injury (56–58).

Protein domain enrichment analysis found that only RNA recognition motif (RNP domain) was enriched in up-regulated DPPs, which was closely related to inflammatory regulation (59). As one of the important domains mediating the interaction between proteins, PDZ domain is enriched in down-regulated DPPs, participating in various biological processes such as intracellular transport, ion channels, and various signal transduction pathways, and its down-regulation can reduce the binding of inflammatory signals (60).

GO cluster analysis showed that DPPs were involved in the regulation of protein unfolding and polymerization. Cellular components are dominated by actin-based cell projection clusters, focal adhesions, cell-substrate junctions, etc. Analysis of molecular function revealed association with mRNA binding. Cluster analysis of KEGG pathway showed that most of the phosphorylated proteins were related to RNA transport, and the down-regulated proteins were related to the regulation of tight junction pathway and actin cytoskeleton. Our experimental results showed that E-selectin, VEGFA, ICAM1 and NGAL were significantly decreased after HUMSCs intervention (**Supplementary Figure 5**), and the expression of endothelial vascular growth factor VEGFA was closely related to tight junction pathway and focal adhesion pathway. VEGF is a key factor in the increased permeability of inflammation-related capillaries in patients with sepsis. VEGF can phosphorylate and dissociate intercellular connection-type molecules, such as vascular endothelial adhesion proteins, thereby promoting endothelial leakage. It is suggested that HUMSCs may regulate the phosphorylation of VEGFA through tight junction pathway or focal adhesion pathway, improve the permeability of endothelial barrier in lung tissue, and alleviate inflammatory injury.

We selected the top 50 phosphorylated proteins with the closest interaction and generated a network containing a total of 173 related phosphorylated proteins. There are many interactions between phosphorylated proteins, which can be considered as pivotal phosphorylated proteins according to the degree score. G3V6K6 (EGFR) found in this study has the most interactions (29 nodes), suggesting that it may be involved in the repair process of septic lung injury. EGFR is widely distributed on the cell surface, and this signaling pathway plays an important role in cell growth, proliferation, and differentiation (61). It has been reported that the inhibition of EGFR can improve the organ dysfunction induced by sepsis (62, 63). Therefore, it is suggested that G3V6K6 (EGFR) may be involved in the repair process of septic lung injury.

Protein phosphorylation is regulated by protein kinases (PKs), and different PKs prefer specific substrates with conserved motifs (64). We performed a bioinformatic analysis using the phosphorylation sites found in this study to identify novel phosphorylation motifs. We performed intensive sequence analysis of hyperphosphorylated site motifs around the phosphate groups of serine and threonine residues using the Motify-X program.

Among the 1316 phosphorylation sites, we identified 123 conserved motifs based on phosphorylated serine (pS) phosphorylation sites and 17 conserved motifs based on phosphorylated threonine (pT) phosphorylation sites. Heat maps were generated showing enrichment or depletion of specific amino acids near the serine phosphorylation site. D, E, P, R and S have a tendency to move to the vicinity of the serine phosphorylation site. P, R, S are largely represented in the proximal region of the threonine phosphorylation site. These amino acids near the phosphorylation sites preferentially reflect the specific recognition of the enzymes that catalyze phosphorylation in septic lung tissue after HUMSCs intervention. Further studies are needed to investigate whether different types of enzymes and kinases are active in regulating phosphorylation.In conclusion, from the perspective of protein phosphorylation modification, we speculate that HUMSCs may regulate the phosphorylation of VEGFA through EGFR-mediated tight junction pathway, improve the permeability of endothelial barrier in lung tissue and alleviate inflammatory injury.

## Conclusion and prospect

Based on proteomics and phosphorylation modification analysis, it was confirmed that HUMSCs intervention could alleviate septic lung injury by regulating specific proteins or phosphorylation modification of proteins, and its basic molecular characteristics were described. Proteomic analysis revealed that TNC may be the key DEPs for HUMSCs to promote the repair of lung injury in juvenile septic rats. HUMSCs may activate TNC-mediated PGE2 release through ECM receptor interaction pathway, improve endothelial cell functional barrier, and promote the recovery of gas-blood barrier function in lung tissue. From the perspective of protein phosphorylation modification, it is suggested that HUMSCs may regulate the phosphorylation of VEGFA through EGFR tight junction pathway, improve the permeability of endothelial barrier in lung tissue, and alleviate inflammatory injury. Results from proteomics and phosphorylated proteomics, the potential new therapeutic targets of HUMSCs in alleviating lung injury in juvenile septic rats were revealed.

However, there are also limitations. Our study is limited to bioinformatics analysis, and the key differential proteins, signaling pathways and phosphorylation sites have not been further verified. In the next step, proteins with significant differences in protein expression or modification levels will be selected for validation and research.

## Data availability statement

The datasets presented in this study can be found in online repositories. The names of the repository/repositories and accession number(s) can be found in the article/**Supplementary Material**.

## Ethics statement

The acquisition and culture of human umbilical cord tissue was approved by the Ethics Committee of the Second Affiliated Hospital of Medical College of Shantou University (Ethics Approval No.: 2021-89). The patients/participants provided their written informed consent to participate in this study. The animal experiment was approved by the Ethics Committee of Shenzhen Topu Biological Laboratory Animal Center (Ethics Approval No.: TOP-IACUC-2021-0003).

## Author contributions

HW and JL: Writing- Original draft preparation, Visualization, Software. AL: experimental design. XS: Methodology. CF: Methodology, Visualization, Software. LX, YW and FW: Writing – review & editing. YL, TW, YZ and LM: Supervision. All authors contributed to the article and approved the submitted version.

## Funding

This research was funded by grants from the National Natural Science Foundation of China (No. 81671525 and No. 8107047), Shenzhen Key Projects of Basic Research (No. JCYJ20200109150618539), Science and technology projects of Guangdong Province (No. 2020-53-112 and No. 2021-88-34), National Science and Technology Major Project (No. 2017ZX09304029004), and Promote special projects for emergency research for epidemic prevention and control technology of COVID-19 of science and technology bureau in Dongguan, China(No. 202071715032116).

## Conflict of interest

The authors declare that the research was conducted in the absence of any commercial or financial relationships that could be construed as a potential conflict of interest.

## Publisher’s note

All claims expressed in this article are solely those of the authors and do not necessarily represent those of their affiliated organizations, or those of the publisher, the editors and the reviewers. Any product that may be evaluated in this article, or claim that may be made by its manufacturer, is not guaranteed or endorsed by the publisher.

## Abbreviation

VEGFA, Vascular Endothelial Growth Factor A; ICAM1, Intercellular Adhesion Molecule 1; HUMSCs, Human Umbilical Cord Mesenchymal Stem Cells; DEPs, Differentially Expressed Proteins; DDPs, Differentially Phosphorylated Proteins; GO, Gene Ontology; KEGG, Kyoto Encyclopedia of Genes and Genomes; PPI, Protein Protein Interaction; PKs, Protein Kinase; ECM, Extracellular matrix; TNC, Tenascin-C; MIF, Macrophage migration Inhibitory Factor; PEG2, Prostaglandin E2; EGFR, Epidermal Growth Factor Receptor; mTOR, Rapamycin; pS, Phosphorylated Serine; pT, Phosphorylated Threonine; vWF, von Willebrand Factor; HMG-Box, High Mobility Group Box; CEs, Carboxylesterases; SEV, Small Extracellular Vesicles; MAPK, Mitogen Activated Protein Kinase; ALI, acute lung injury; UPLC, Ultra Performance Liquid Chromatography.
