# Supplementary material for: Proteomic and phosphorylated proteomic landscape of injured lung in juvenile septic rats with therapeutic application of umbilical cord mesenchymal stem cells
Source: Front Immunol. 2022 Oct 21;13:1034821. doi: 10.3389/fimmu.2022.1034821 (PMC9635340; doi:10.3389/fimmu.2022.1034821)
Supplement: Supplementary file 1 [file DataSheet_1.doc]

**SUPPLEMENTARY MATERIALS**

**Supplementary Table 1 Reagents commonly used in this study**

| **Reagent name** | **Reagent manufacturer** |
| --- | --- |
| H₂O | Fisher Chemical |
| Protease Inhibitor Cocktail III | Merck Millipore |
| Protease Inhibitor Cocktail V | Merck Millipore |
| Protease Inhibitor Cocktail VI | Merck Millipore |
| Protease Inhibitor Cocktail IV | Merck Millipore |
| trypsin | Promega |
| Phosphorylase inhibitor | Millipore |
| iodoacetamide, IAM | Sigma-Aldrich |
| formic acid | Fluka |
| urea | Sigma-Aldrich |
| DL-Dithiothreitol, DTT | Sigma-Aldrich |
| Acetonitrile | ThermoFisher Scientific |
| trifluoroacetic acid, TFA | Sigma-Aldrich |
| Tetraethylammonium bromide, TEAB | Sigma-Aldrich |
| BCA kit | Beyotime Biotechnology |
| 2-D Quant kit | GE Healthcare |
| Methanol | ThermoFisher Scientific |
| ethanol | Gaojing Chemical |
| dimethylbenzene | Sangon Biotech |
| DNA Phenol extraction reagent | Solarbio |
| AceticAcid | Gaojing chem |
| coomassie brilliant blue | Beyotime Biotechnology |
| Acetone | Hangzhou Hanno Chemical |
| Trichloroacetic acid,TCA | Sigma-Aldrich |
| Ethylene Diamine Tetraacetic Acid, EDTA | Sigma-Aldrich |
| 2-Hydroxy-1-ethanethiol | Sigma-Aldrich |
| Sodium dodecyl sulfate, SDS | Amresco |
| Ammonium bicarbonate | Sigma-Aldrich |
| Triton X-100 | Sigma Sangon Biotech |
| Glycine | Amresco |
| Trichloromethane | Sigma-Aldrich |
| Acrylamide | Sigma-Aldrich |
| Tris-base | Sigma-Aldrich |
| ammonium acetate | Sinopharm Chemical Reagent |
| PR-619 | Selleck Chemicals |
| Trichostatin A ，TSA | MedChemExpress |
| Nicotinamide, NAM | Sigma-Aldrich |
| TMT labeling reagent | ThermoFisher Scientific |
| ammonium persulfate, APS | Sangon Biotech |
| hydrochloric acid | Sigma-Aldrich |
| N,N,N,N-Tetramethylethylenediamine,TEMED | Sigma-Aldrich |
| Sodium chloride | Sigma-Aldrich |
| Potassium chloride | Sigma-Aldrich |
| Potassium dihydrogen phosphate | Sigma-Aldrich |
| Dibasic Sodium Phosphate | Aladdin |
| agarose gel | Sangon Biotech |
| Pierce Top 12 Abundant Protein Depletion Spin Columns | Thermo scientific |
| Proteominer™ Protein Enrichment Small-Capacity Kit | Bio-Rad Laboratories |
| Seppro ® Rat Spin Columns | Sigma-Aldrich |
| D(+)-Sucrose | Sinopharm Chemical Reagent |
| PAGE silver staining kit | Solarbio |
| Glycerol | Sangon Biotech |
| Ponceau S | Amresco |
| Protein Marker | Thermo scientific |
| milk powder | Amresco |
| Mouse IgG (H+L) Secondary Antibody | Pierce |
| Rabbit IgG (H+L) Secondary Antibody | Pierce |
| nitrocellulose filter membrane, NC | Bio-Rad Laboratories |
| polyvinylidene fluoride, PVDF | Bio-Rad Laboratories |
| Horseradish Peroxidase, HRP | Millipore |

**Supplementary Table** 2 The top 20 up-regulated and down-regulated DEPS

| **Proteins** | **Gene** | **Ratio** | **P value** | **Regulation type** |
| --- | --- | --- | --- | --- |
| Q3B7U1 | Maged2 | 7.4986 | 0.013673629 | Up |
| Q499S4 | Mx1 | 4.5891 | 0.030959646 | Up |
| Q923W4 | Hdgfl3 | 3.8488 | 0.009829112 | Up |
| D3ZCI9 | Myl10 | 3.7722 | 0.003230482 | Up |
| Q6AYA7 | Rfk | 3.7153 | 0.00142478 | Up |
| D3ZCR3 | LOC108349189 | 3.5128 | 0.000373895 | Up |
| B0K034 | Dtd2 | 3.3921 | 0.000645117 | Up |
| A0A0G2JT86 | -- | 3.3047 | 0.000879847 | Up |
| G3V6T2 | LOC103689922 | 3.2912 | 0.03665022 | Up |
| Q5FVL6 | Tspan13 | 3.1222 | 0.009648892 | Up |
| P52925 | Hmgb2 | 3.0177 | 0.002225335 | Up |
| D3ZK56 | Rap2c | 2.9481 | 0.000123245 | Up |
| Q9WVI4 | Gucy1a2 | 2.8904 | 0.001437469 | Up |
| A0A0G2K1L0 | Tnc | 2.8903 | 0.003247663 | Up |
| A0A0G2JTA7 | Rasal2 | 2.8772 | 0.001334604 | Up |
| B0BNL6 | Arrdc1 | 2.8117 | 5.23646E-05 | Up |
| G3V726 | Gzmm | 2.8092 | 0.011131462 | Up |
| D4A2I4 | Ormdl2 | 2.8081 | 0.000102987 | Up |
| Q920R3 | Fads1 | 2.7784 | 0.000502834 | Up |
| A0A0G2K500 | Npnt | 0.4989 | 0.003035408 | Down |
| F1M9Y9 | Rbpms | 0.4984 | 7.97777E-05 | Down |
| D4A3T3 | Cbx1 | 0.4982 | 0.000140074 | Down |
| F7F350 | Syap1 | 0.4961 | 0.00632715 | Down |
| A0A0G2KAW7 | Eif4h | 0.4953 | 0.003540304 | Down |
| Q68FX0 | Idh3B | 0.4929 | 0.002181131 | Down |
| Q62952 | Dpysl3 | 0.4923 | 0.021301179 | Down |
| F1LRC2 | Aif1 | 0.4886 | 0.001098109 | Down |
| Q62658 | Fkbp1a | 0.4885 | 0.025088178 | Down |
| P30904 | Mif | 0.4862 | 0.008183515 | Down |
| P61972 | Nutf2 | 0.485 | 0.041570247 | Down |
| Q4V7D1 | Ssr1 | 0.4844 | 0.001264553 | Down |
| P55053 | Fabp5 | 0.4836 | 0.045262885 | Down |
| Q6TXF3 | Dbi | 0.4823 | 0.032033455 | Down |
| A0JPM9 | Eif3j | 0.4819 | 0.016371715 | Down |
| Q63362 | Ndufa5 | 0.4805 | 0.049232605 | Down |
| P24155 | Thop1 | 0.4801 | 0.029998384 | Down |
| Q5FVR7 | Cpsf4 | 0.4776 | 0.019587023 | Down |
| Q499N6 | Ubxn1 | 0.4776 | 0.008214709 | Down |
| F1M9N7 | Agfg1 | 0.4756 | 0.004332268 | Down |

**Supplementary Table** 3 Top 15 Hub Proteins in PPI Network Based on Expression Degree

| **protein accession** | **Degree** | **Gene name** | **Regulated Type** | **Ratio** | **P value** |
| --- | --- | --- | --- | --- | --- |
| P06399 | 9 | Fga | Down | 0.4277 | 0.017256172 |
| G3V9M6 | 9 | Fbn1 | Down | 0.2993 | 0.015282018 |
| D3ZFH5 | 8 | Itih2 | Down | 0.4171 | 0.00989387 |
| D4A6W6 | 8 | -- | Down | 0.3702 | 0.007758394 |
| P62832 | 8 | Rpl23 | Down | 0.245 | 0.042871697 |
| A0A0H2UHQ8 | 8 | Rps17 | Down | 0.3268 | 0.004226078 |
| A0A0H2UHH9 | 8 | Rps24 | Down | 0.3667 | 0.00075022 |
| A0A0H2UH99 | 8 | Rpl24 | Down | 0.4175 | 0.017564335 |
| P62278 | 8 | Rps13 | Down | 0.1189 | 0.000191713 |
| P21533 | 7 | Rpl6 | Down | 0.4659 | 0.00182108 |
| B5DEG1 | 7 | Itga8 | Down | 0.4722 | 0.001799768 |
| P62912 | 7 | Rpl32 | Down | 0.2687 | 0.004960199 |
| P01048 | 7 | Map1 | Down | 0.4009 | 0.0413995 |
| Q80W89 | 6 | Ndufa11 | Down | 0.3446 | 0.014592745 |
| A0A0G2K1L0 | 6 | Tnc | Up | 2.8903 | 0.003247663 |

**Supplementary Figure 1: Intraperitoneal injection of cecal contents to establish juvenile septic rat**

About 4 hours after intraperitoneal injection of cecal content solution, 4-week-old SD rats have poor mental state, erect hair, accelerated breathing, curled up body, decreased vitality, reduced intake of water and slow response to stimulation, suggesting that pediatric rats have clinical manifestations of sepsis. Fifteen 4-week-old SD rats were intraperitoneally injected with cecal contents. 9 rats died within the observation time (72 hours), and the mortality was 60%. (Supplementary Fig.1a). Compared with the normal group, leukocytes, neutrophils and lymphocytes of rats with sepsis symptoms after intraperitoneal injection of cecal contents decreased significantly (P < 0.05), began to increase at 24 hours (P < 0.05), and monocytes decreased when pediatric rats had sepsis symptoms (P < 0.05), and began to rise at 9 hours. Platelets remained lower than normal level after the symptoms of sepsis in pediatric rats until the end of the observation period (P < 0.05) (Supplementary Fig.1b). Compared with the normal group, the expression of CRP, SAA, E-selectin, VEGFA, ICAM1 and NGAL at each time point after intraperitoneal injection of cecal content increased significantly (P < 0.05).and PGE2 decreased (P < 0.05) (Supplementary Fig.1c). After sepsis symptoms occurred in juvenile rats after intraperitoneal injection of cecal contents, HE staining of lung tissue showed thickening of alveolar wall, accompanied by granulocyte infiltration and falling cell fragments in local bronchus. 24 hours after the onset of sepsis symptoms occurred, HE staining of liver tissue showed that granular degeneration, loose cytoplasm and light staining of hepatocytes were widely seen around the central vein and portal area and in the liver parenchyma (Supplementary Fig.1d).


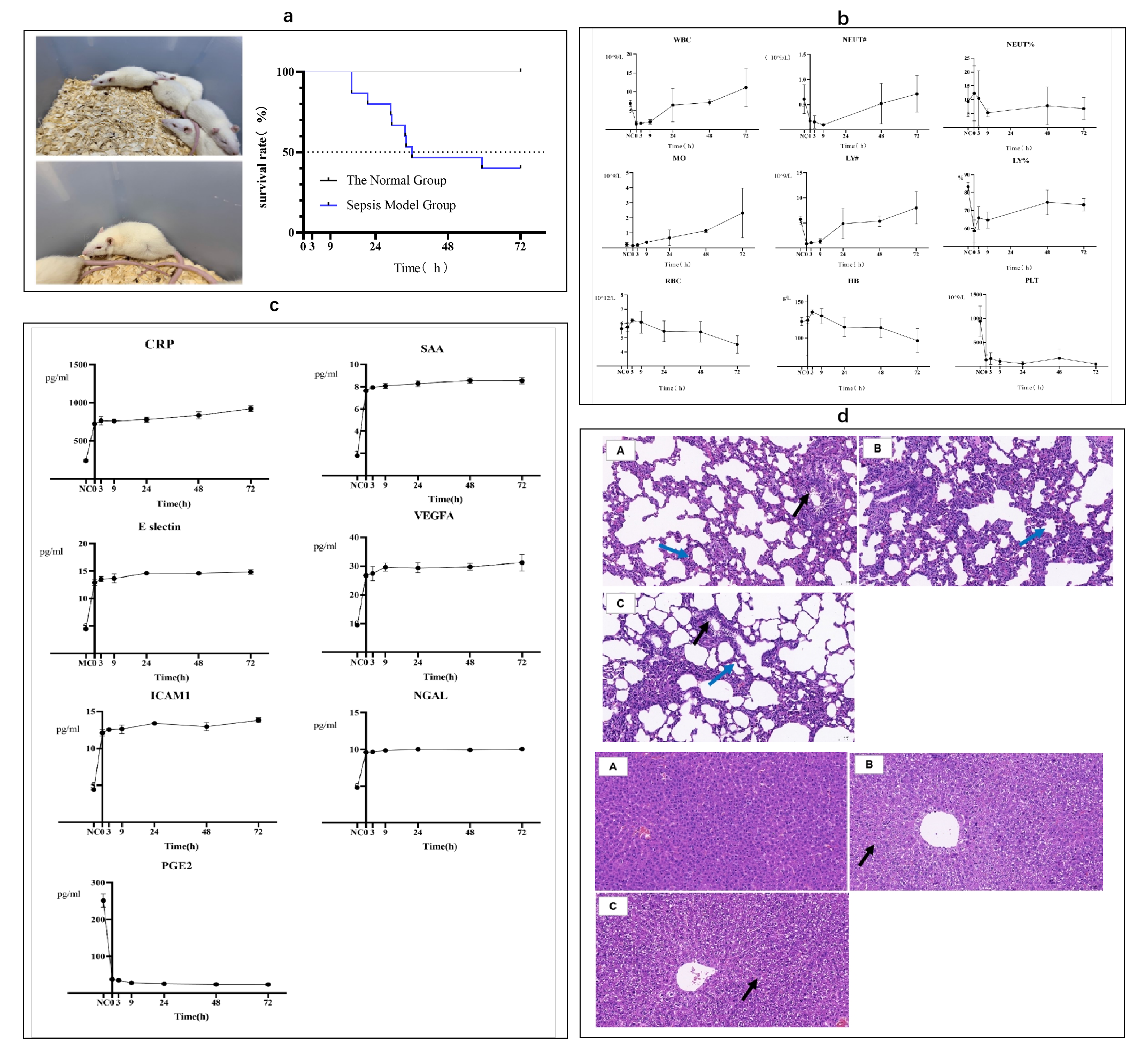


Supplementary Figure 1 Intraperitoneal injection of cecal contents to establish juvenile septic rat

SD rats were intraperitoneally injected with cecal contents showed clinical manifestations of sepsis, and the mortality rate reached 60% after 72 hours of observation (a); SD rats were intraperitoneally injected with cecal contents the changes of blood routine parameters at different time points (b); SD rats were intraperitoneally injected with cecal contents the changes of blood inflammatory markers and endothelial cytokines at different time points (c); Lung and liver injury in SD rats were intraperitoneally injected with cecal contents (d).

**Spplementary Figure 2: Culture, Amplification and Identification of HUMSCs**

When human umbilical cord tissue mass was incubated in mesenchymal stem cell culture medium for 5-7 days, it can be seen that some tissue mass adhered to the wall and became round, and new cells crawled out around it, showing spindle shape and sparse distribution (Supplementary Fig.2a). After incubating for 7-10 days, the density of primary cells increased obviously, and tended to fuse. The cells were slender and enlarged in size, similar to fibroblasts. The growth rate of primary cells is slow, about 10-14 days, and the fusion degree of cells reaches 80%-90%, which can be passed. After passage, the cells grew rapidly, with 1: 3 passage. In this experiment, the third generation cells were used. At this stage, the cells were long spindle-shaped and had good growth vitality (Supplementary Fig.2b). Eight surface markers of MSCs were selected as the surface markers for detecting HUMSCs, which were CD 73, CD 90, CD 105, CD 11B, CD 34, CD 45 and HLA-DR. In this experiment, the surface markers of MSCs were selected from the third generation cells. The results showed that HUMACs stably expressed CD73 (99.01%), CD90 (95.6%) and CD105 (96.2%), and expressed CD11B (0.80%), CD19 (0.6%), CD34 (0.4%), CD45 (0.6%) and HLA-DR (0.6%) (Supplementary Fig.2c).


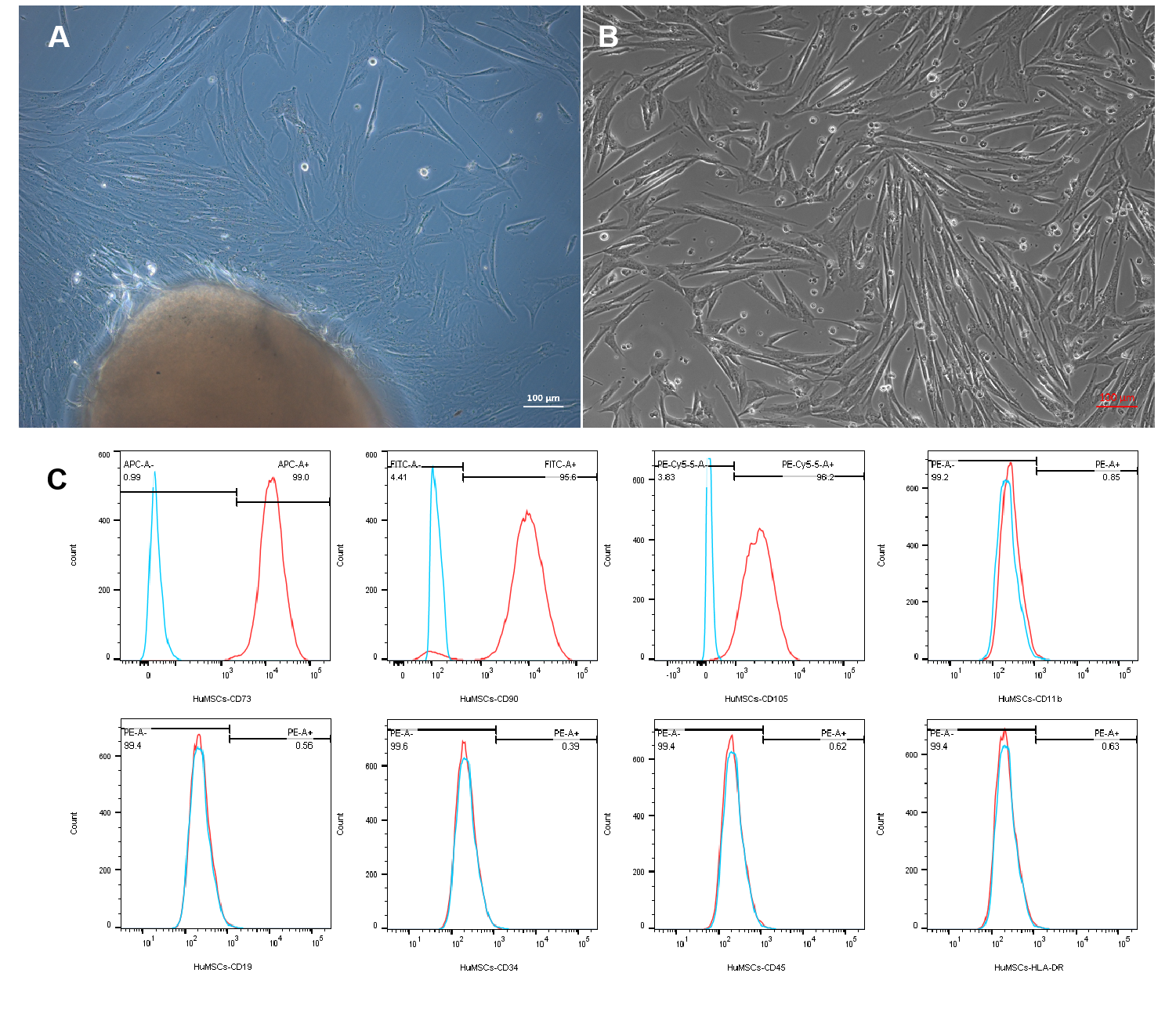


Supplementary Figure 2 Culture and Identification of HUMSCs

The umbilical cord tissue mass adhered to the wall, and new cells crawled out and fused with each other (a); The third generation cells are long spindle-shaped and have good growth vitality (b); Flow phenotype of HUMSCs (c).

**Supplementary Figure 3: Pathological morphology of lung tissue of juvenile septic rats after intervention with HUMSCs**

Lung histopathology in the sepsis model group showed slight thickening of the alveolar wall with granulocyte infiltration (blue arrow), and a small amount of exfoliated cell debris (black arrow) in the local bronchi (Supplementary Fig. 3a, b). Compared with the sepsis model group, 24 hours after the intervention of HUMSCs, the bronchial epithelial cells were closely arranged, occasionally the epithelial cells were slightly swollen, the cytoplasm was loose and lightly stained (black arrow), the alveolar wall was thickened in a large area (red arrow), a small number of inflammatory cells on the alveolar wall were scattered and infiltrated (yellow arrow), the alveoli were clean, and no other obvious abnormalities were found in the tissue (Supplementary Fig. 3c); Pathology of the lung tissue 48 hours after intervention by HUMSCs showed that the bronchial epithelial cells were closely arranged, occasionally the epithelial cells were slightly swollen, the cytoplasm was loose and lightly stained (black arrow), the alveolar wall was thickened in a large area (red arrow), a small number of inflammatory cells on the alveolar wall were scattered and infiltrated (yellow arrow), the alveoli were clean, and no other obvious abnormalities were found in the tissue (Supplementary Fig. 3d).


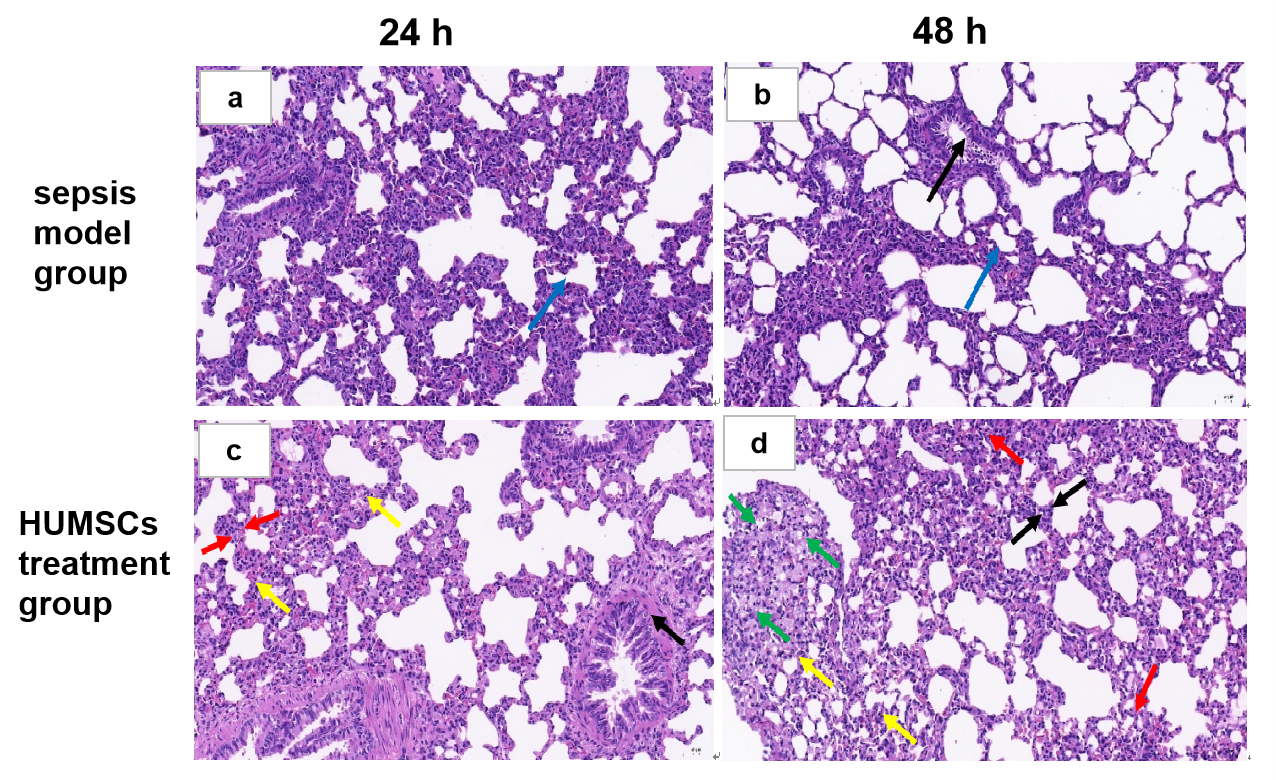


Supplementary Figure 3 Pathological morphology of lung tissue of juvenile septic rats after intervention with HUMSCs for 24 hours and 48 hours was significantly improved compared with the sepsis group without intervention, (20 ×).

Lung histopathological changes of sepsis model group without intervention for 24 hours (a). Lung histopathological changes of sepsis model group without intervention for 48 hours (b). Pathological morphology of lung tissue of juvenile rats with sepsis intervened by HUMSCs for 24 hours (c). Pathological morphology of lung tissue of juvenile rats with sepsis intervened by HUMSCs for 48 hours (d).

**Supplementary Figure 4 Ultrastructural changes of lung gas-blood barrier in juvenile septic rats after treatment with HUMSCs**

The ultrastructure of gas-blood barrier in lung tissue of sepsis model group (0 hour) showed that the cytoplasm of capillary endothelial cells (end) was edema, mainly intracellular matrix edema, and the tight junction (Tj) between cells was slightly blurred. Basement membrane (BM) is intact and continuous. I (type I epithelium) The cytoplasm of the epithelial cells continuously covers the alveoli (AV). The epithelial cells are severely edematous and the local cell membrane is damaged. Endothelial cell nucleus (N) was oval, heterochromatin was a little more, part of the nuclear membrane was blurred; mitochondria (M) was obviously swollen, the matrix in the membrane became pale, and the cristae were broken and reduced. The rough endoplasmic reticulum (RER) was significantly dilated and the membrane was damaged; there were more red blood cells (RBC) in the capillary lumen, and the lumen was embolized (Supplementary Fig.4 a, b).

The gas-blood barrier ultrastructure of lung tissue in sepsis model group (24 hours) showed that the cytoplasm of capillary endothelial cells (end) was edema, the overall structure was acceptable, the cell membrane was intact, and the number of intercellular tight junctions (Tj) was large. Basement membrane (BM) is complete, continuous and uniform in thickness. I (type I epithelium) The cytoplasm of the epithelial cells continuously covers the alveoli (AV), which is flat, the cytoplasm is edematous, and the electron density is reduced. Endothelial cell nucleus (N) was irregular, heterochromatin was slightly more, and the nuclear membrane was indistinct; the structure of mitochondria (M) was acceptable, no obvious swelling was found, the membrane was intact, and cristae existed. The rough endoplasmic reticulum (RER) was slightly dilated, the Golgi apparatus (Go) was normal, and there was no obvious hyperplasia and hypertrophy; the capillary lumen was not obviously shrunken and collapsed, and there was a small amount of red blood cells (RBC) in it, and the alveolar space (AV) had myelin sheath-like and free floccules (Supplementary Fig.4 c, d).

24 hours after the treatment of HUMSCs, the capillary endothelial cells (Enc) showed significant pyknosis in individual and apoptosis trend in the whole, with intact cell membrane, high electron density in the cells, slightly swollen organelles and a large number of tight junctions (TJ) between cells. Basement membrane (BM) is complete, continuous and uniform in thickness. The nucleus (N) was irregular in shape, with obvious pyknosis, increased heterochromatin, blurred nuclear membrane and widened perinuclear space; the mitochondria (M) was slightly swollen with intact membrane. The rough endoplasmic reticulum (RER) was dilated and degranulated, and a small amount of autophagic lysosomes (ASS) was present; the capillary lumen was not significantly wrinkled and collapsed, and a small amount of red blood cells (RBC) were present (Supplementary Fig. 4 e, f).


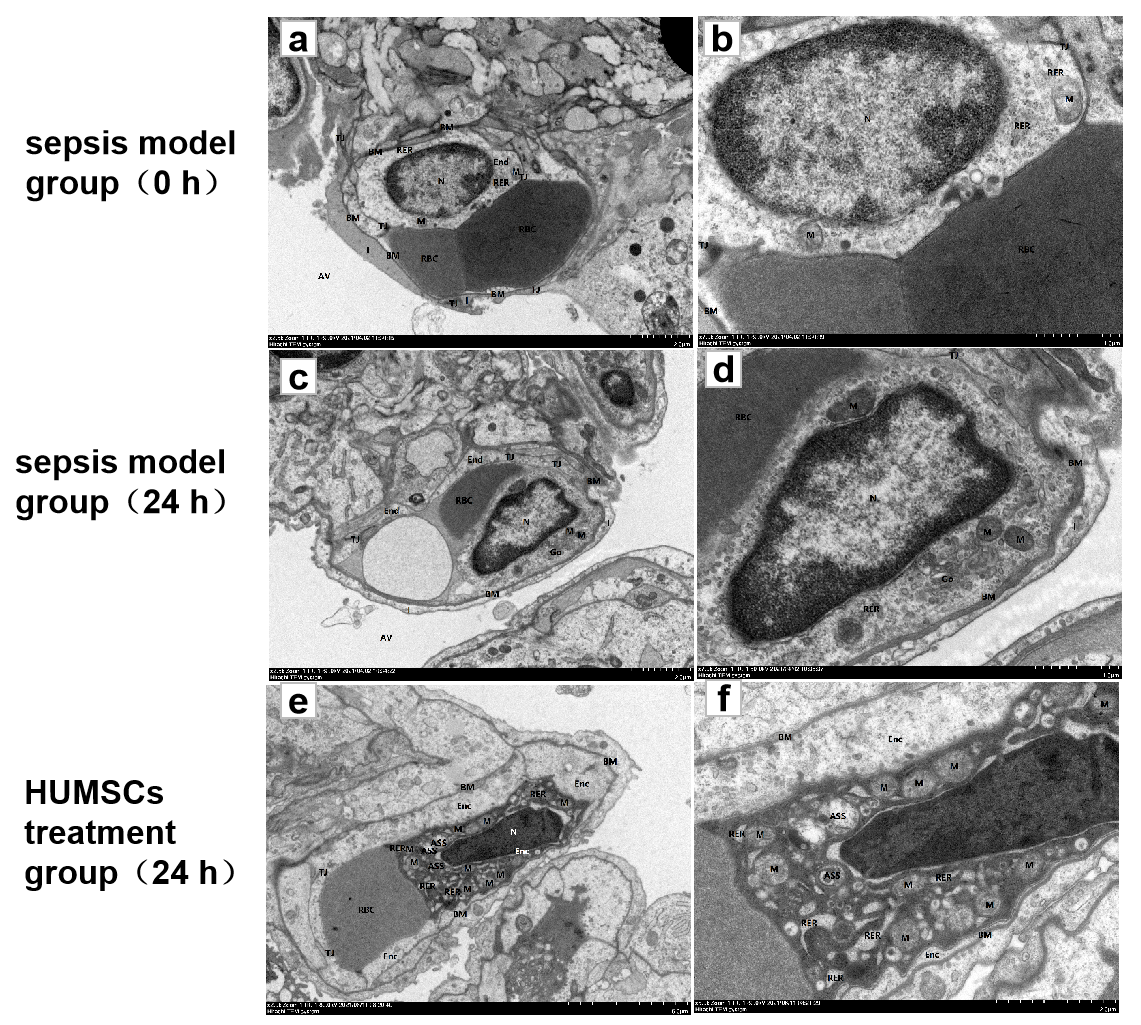


Supplementary Figure. 4 The ultrastructure of lung gas-blood barrier in sepsis model group was significantly damaged, and the damage of lung gas-blood barrier-capillary was alleviated 24 hours after treatment with HUMSCs.

Ultrastructural changes of lung tissue gas-blood barrier in sepsis model group (0 hour) (a and b); ultrastructural changes of lung tissue gas-blood barrier in sepsis model group (24 hours) (c and d); ultrastructure changes of lung tissues gas-blood barrier after intervention of HUMSCs for 24 hours (e and f).

**Supplementary Figure 5 Changes of serum endothelial cytokines in juvenile septic rats after HuMSCs intervention**


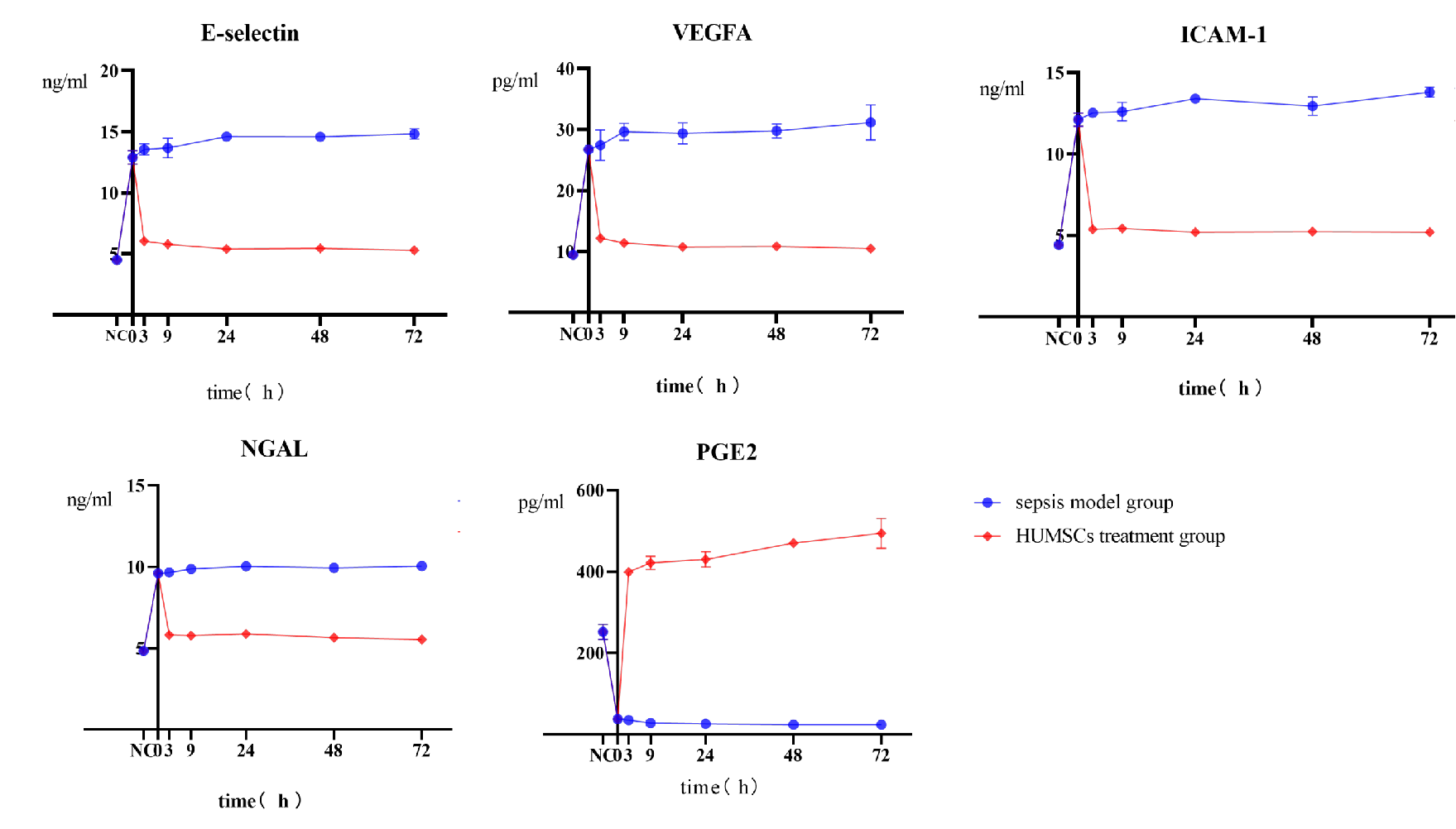


Supplementary Figure 5 Compared with sepsis model group, the expression of E-selectin, VEGFA, ICAM-1, NGAL at 3 hours, 9 hours, 24 hours, 48 hours, 72 hours after HUMSCs intervention decreased significantly, but the expression of PGE2 increased significantly (P < 0.05).

**Supplementary Figure 6 Tight junction pathway map**

**
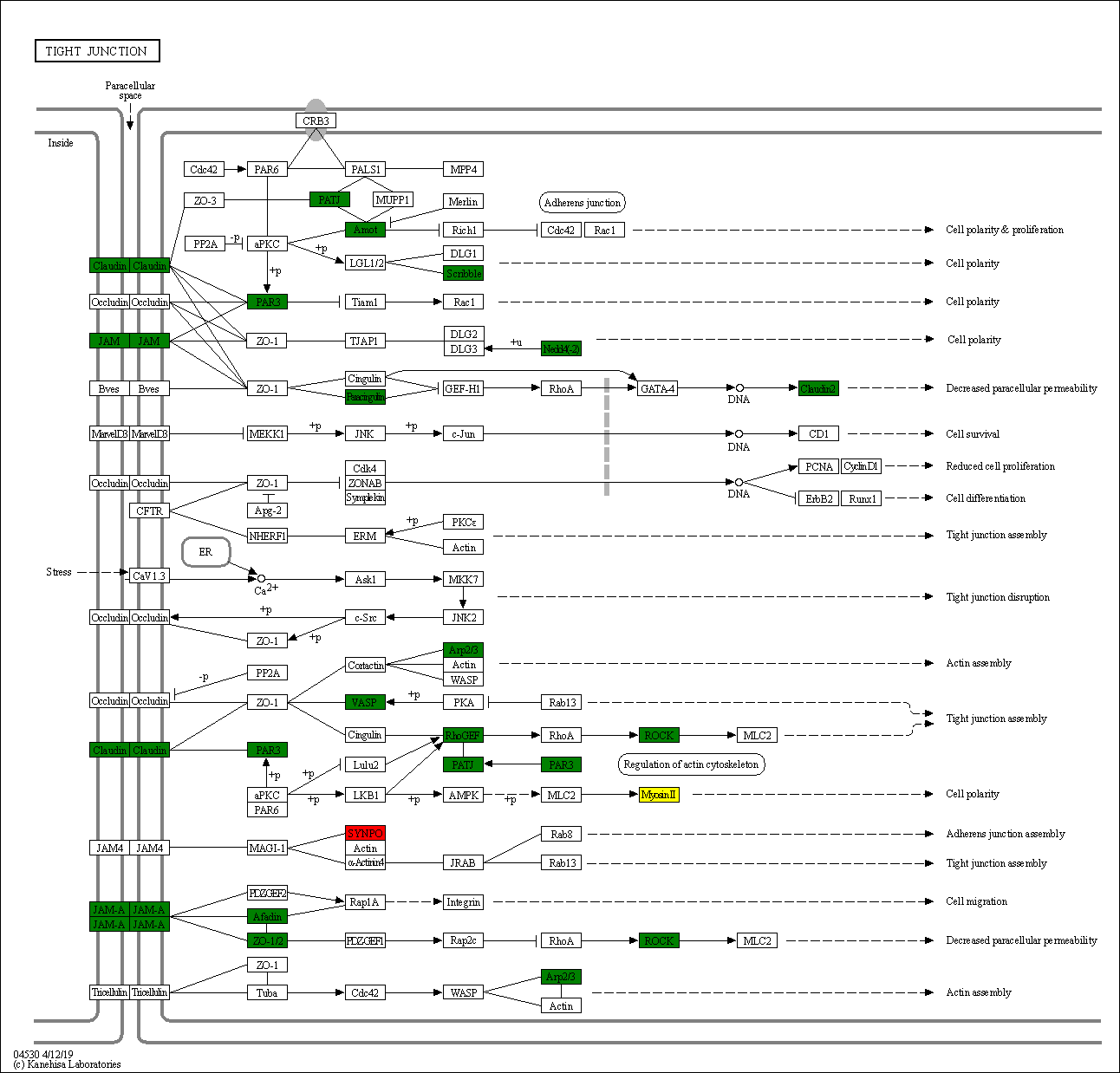
**

**Supplementary Figure 6** Tight junction pathway map. Tight junctions encode genes for epithelial intercellular junction proteins, indicating that there is interference in the permeability between epithelial barriers, which may be closely related to the regulation of intercellular barriers in septic lung injury.
